# A Data Driven Review of In Vitro Electrical and Mechanical Stimulation for Post‐Acute Phase Wound Healing

**DOI:** 10.1002/adhm.71138

**Published:** 2026-05-02

**Authors:** Matthew K. Burgess, Ellen F. Marsh, Veronica M. Lucian, Malavika Nair

**Affiliations:** ^1^ Institute of Biomedical Engineering, Department of Engineering Science University of Oxford Oxford UK

**Keywords:** electrical stimulation, fibroblasts, keratinocytes, mechanical stimulation, wound healing

## Abstract

This review explores the role of in vitro electrical and mechanical stimulation in modulating wound‐healing behavior, with a primary focus on the predominant skin cell types: fibroblasts and keratinocytes. By analyzing the existing literature, we delineate the complex relationships between stimulation parameters—such as voltage, current, frequency, and mechanical strain—and cellular responses, including proliferation and migration. Our data‐driven approach compiled more than 390 experimental data points for electrical stimulation and over 170 for mechanical stimulation in vitro, constructing a comprehensive library of cell responses that were previously fragmented and difficult to compare across studies. We critically evaluate various stimulation platforms and configurations, emphasizing their influence on cellular mechanobiology and their translational potential in regenerative medicine. Ultimately, this review underscores the necessity of a multi‐parameter optimization strategy to effectively exploit electromechanical cues for targeted skin tissue regeneration.

## Graphical abstract

This review examines how in vitro electrical and mechanical stimulation modulates wound healing in fibroblasts and keratinocytes. Analyzing over 560 experimental data points, we relate stimulation parameters to proliferation and migration outcomes, evaluate platform designs, and highlight the need for multi‐parameter optimization to advance targeted skin regeneration strategies.

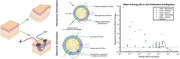

## 1 Introduction

The primary objective of wound healing is to regenerate healthy tissue at sites of injury and maintain organ function. As the largest organ in the body and the main barrier to the external environment, the skin plays a critical role in thermoregulation, mechanical protection, hydration control, and sensory detection [1]. These functions arise from the highly organised arrangement of cells embedded within a layered extracellular matrix (ECM). The ECM, composed predominantly of collagen (50%–90%) and elastin (0.6%–7.9%), governs the mechanical integrity of skin tissue [2]. The distribution and alignment of these fibers determine stress concentrations within the matrix, while the overarching tensegrity of the network translates external forces into strain fields within the cellular microenvironment.

In addition to these mechanical systems, biochemical and bioelectric signaling are equally vital in maintaining tissue homeostasis. Endogenous bioelectric fields arise from ion transport and cellular membrane potentials that regulate numerous physiological processes, including cell migration, proliferation, and wound closure [3]. These intrinsic electrical cues form part of the bioelectric network of the body, acting in concert with biochemical and mechanical signals to sustain tissue integrity. Collectively, these interconnected systems sustain the homeostatic environment required for cellular survival. Disruption to any component of this feedback‐mediated regulatory network can compromise the structural and functional integrity of the tissue, initiating the wound healing cascade.

Wound healing follows a sequence of overlapping stages, each designed to mitigate risk to life and restore tissue function. The immediate response, hemostasis, aims to prevent blood loss through vessel constriction and clot formation [4]. This is followed by the inflammatory phase, wherein damaged ECM releases cytokines and growth factors that recruit macrophages and neutrophils to eradicate infection and remove debris. Once hemostasis and inflammation are established, the wound transitions to the proliferative stage. Fibroblasts, attracted by platelet‐derived growth factor (PDGF) and tissue growth factor (TGF)‐β secreted by inflammatory cells, migrate into the wound bed, proliferate, and synthesize ECM proteins such as collagen [4, 5]. Concurrently, keratinocytes proliferate and migrate across the wound surface to re‐epithelialize the tissue, while endothelial cells drive angiogenesis to re‐establish vascular networks. This orchestrated cellular activity forms granulation tissue, a collagen‐rich matrix that provides temporary structural integrity. Over time, this tissue undergoes remodeling, restoring tensile strength and auxiliary skin functions such as sensation and thermoregulation [5].

Deviations from this tightly regulated sequence, particularly in complex or co‐morbid wounds can lead to chronic, non‐healing conditions that require prolonged care and continuous support [6, 7]. Efforts to accelerate wound healing typically aim either to prevent chronic wound development or to reactivate stalled regenerative processes [6, 8]. Conventional strategies rely on pharmacological and biochemical interventions such as growth factors [9], anti‐inflammatory agents [10], and antimicrobial [11] therapies to promote cell proliferation and angiogenesis or control infection [6]. However, these approaches often suffer from limited efficacy and scalability, particularly in complex wound environments.

**FIGURE 1:**
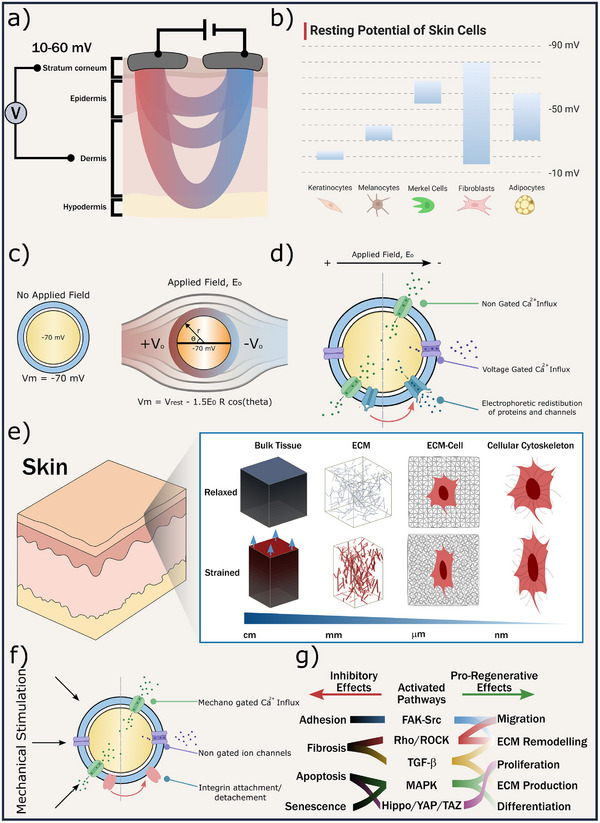
Principles of bioelectromechanics in skin: (a) the inherent trans‐epithelial potential and the application of electric field for a cross‐section of full‐thickness skin. (b) The resting potential of skin cells, with keratinocytes possessing the lowest values (−20 to −30V) [3, 13, 14] and fibroblasts displaying the largest range (−70 to −15 mV) [3, 15, 16, 17]. (c) The influence of electric fields are often modeled using simplified models considering a monolithic cell affected by macroscopic electric fields (d) locally, the asymmetric impact of electric fields induces non‐voltage‐gated calcium influx as a result of increased extracellular calcium concentration towards the cathode, voltage‐gated calcium influx from cathodic activation of channels, and electrophoretic redistribution of proteins and channels due to field exposure. (e) a scale‐dependent view of the impact of mechanical loads on cell behavior, including deformation and strains across the bulk tissue, the extracellular matrix components, the ECM‐Cell interfaces down to the direct stresses applied on cell receptors and cytoskeletal proteins. (f) mechanical loading can not only cause changes in attachment through integrin‐mediated receptors, but can also change ion flux in the cell through both non‐gated ion channels and mechano‐gated Ca^2+^ channels. (g) electrical and mechanical modulation can activate or suppress the FAK‐Src, Rho/ROCK, TGF‐β, MAPK, Hippo/YAP/TAZ pathways, which are directly implicated in the response of fibroblast and keratinocyte migration, proliferation, and differentiation as well as ECM production and remodeling. Image modified from Robinson [18].

Due to the intrinsic electrical and mechanical cues (Fig. 1) that coordinate cell behavior and ECM dynamics, there is growing interest in employing exogenous electromechanical stimulation as a therapeutic strategy for skin regeneration [12]. From in vitro studies that investigate underlying mechanisms to in vivo clinical applications, a substantial body of literature seeks to optimize stimulation parameters for promoting cell migration, proliferation, and matrix remodeling. Yet, the diversity of stimulation modalities and experimental methodologies has produced a heterogeneous set of results, complicating the identification of optimal stimulation regimes. Although the immune system initiates the wound‐healing cascade, the post‐inflammatory transition represents a critical functional inflection point at which the acute inflammatory microenvironment is actively resolved, thereby permitting the onset of tissue reconstruction. This review specifically addresses the subsequent reparative phase, during which the dominant contribution of infiltrating immune cells is dominated by the activities of fibroblasts and keratinocytes as the principal effector cell populations mediating ECM deposition and re‐epithelialization. By focusing on this stage, we aim to delineate the specific patterns of cellular activation and responsiveness that occur once the inflammatory response has peaked and begun to subside.

This review provides a narrative outline of electromechanical stimulation supported by a data‐driven analysis of the current literature on electrical and mechanical stimulation of skin cells, specifically fibroblasts and keratinocytes that dominate post‐inflammatory wound healing processes. In particular, we examine in vitro stimulation set‐ups reported in the literature, to tease out overarching trends in stimulation parameters. Through this combined approach, we aim to highlight how exogenous stimulation influences key cell behaviors to assess its potential for targeted skin regeneration.

## 2 Cellular Response to Electromechanical Cues

### 2.1 Fibroblasts

**FIGURE 2:**
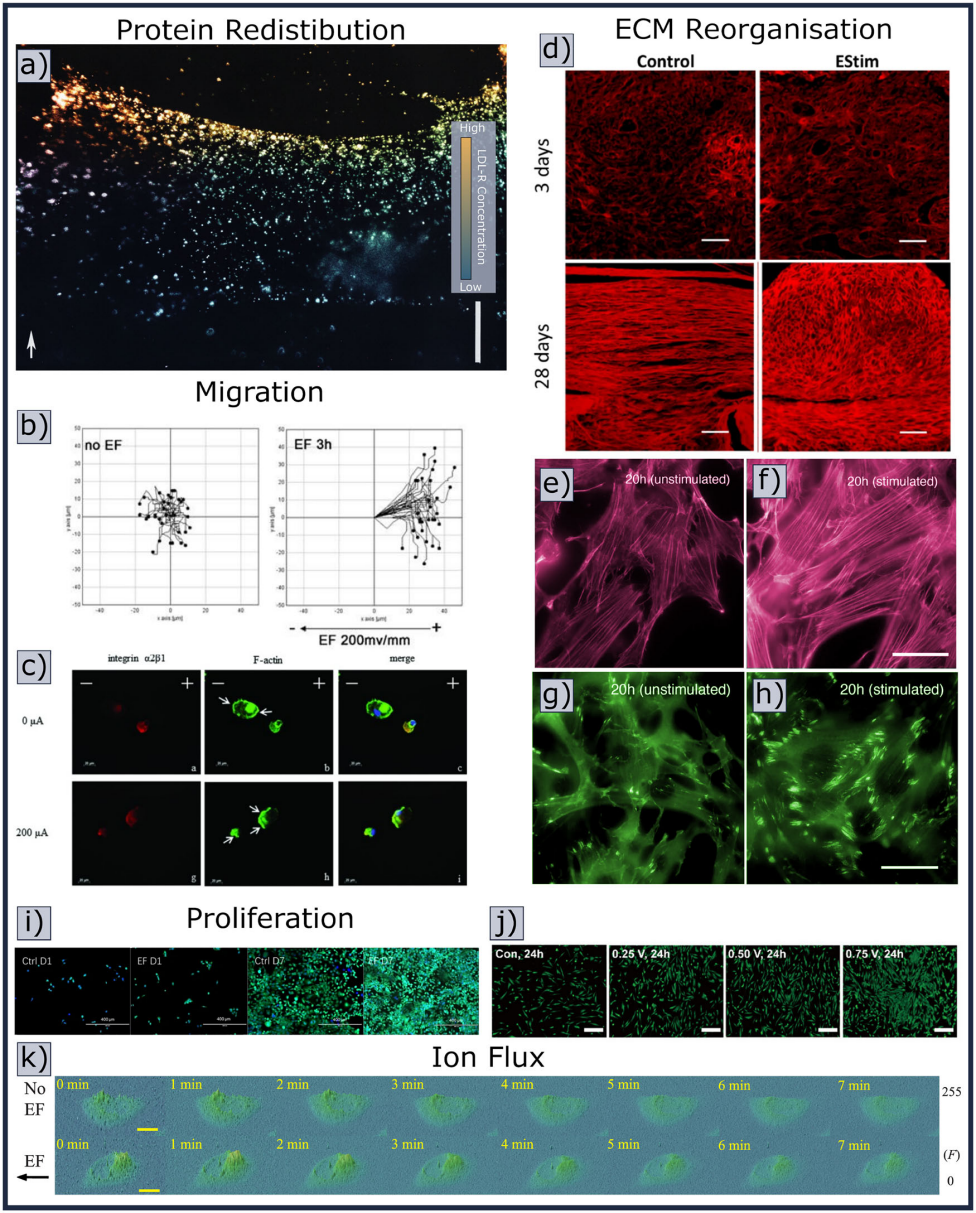
The mechanistic effects of electrical stimulation on fibroblasts and keratinocytes include protein redistribution and ion flux, as well as behavioral alterations such as migration, proliferation and ECM remodeling. (a) Protein redistribution illustrated by a fluorescence micrograph showing electric field–induced redistribution of LDL‐R on a fibroblast; receptors visualized with post‐field‐applied diI(3)‐LDL show an exponential decrease in surface concentration with increasing distance from the cathodal edge (field applied perpendicular to the long cell axis; scale bar 12 μm, adapted from Tank et al. [22]) under CC BY‐NC‐SA 4.0 (b) Migration trajectories of human dermal fibroblasts (HDFs) recorded over 3 h in the absence (left) or presence (right) of a 200 mV/mm electric field, demonstrating increased displacement and directional migration towards the cathode under stimulation, reproduced from Wang et al. [23], under CC BY 4.0 c) Integrin α2β1 polarization and lamellipodia formation in fibroblasts exposed to monophasic pulsed microcurrent; immunofluorescence images show integrin α2β1 (red) and F‐actin (green) at 0 μA (top) and 200 μA (bottom), with lamellipodia and integrin clustering at the field‐facing cell edges in stimulated cells, adapted from Uemura et al. [24], under CC‐BY. (d) ECM reorganization in vivo shown by Picro‐Sirius Red–stained collagen at distal limb stumps in control and electrically stimulated tissue at 3 and 28 days post‐amputation; electrical stimulation alters collagen fiber alignment and increases interfibrillar spacing relative to controls, adapted from Oliveira et al. [25], under CC BY 4.0. (e, f) Actin cytoskeleton remodeling in fibroblasts; stress fibers visualized after 20 h culture without (e) or with (f) periodic electrical stimulation (50 V, 60 pulses/min), where stimulation increases the number and thickness of stress fiberrs (scale bars 20 μm), adapted from Katoh [26], under CC BY 4.0. g–h) Focal adhesion remodeling visualized by paxillin staining in fibroblasts cultured for 20 h without (g) or with (h) periodic electrical stimulation, showing enlarged and more numerous focal adhesions in stimulated cells (scale bars 20 μm), adapted from Katoh [26], under CC BY 4.0. (i) Effect of electric fields on keratinocyte proliferation and viability: Hoechst and Calcein‐AM staining of the immortal keratinocyte cell line from adult human skin (HaCaT cells) exposed to a 200 mV/mm field, illustrating increased numbers of viable cells over culture time compared with unstimulated controls, adapted from Lu et al. [27], under CC BY 4.0. (j) Proliferation of human gingival fibroblasts (HGFs) 24 h after electrical stimulation, visualized by calcein‐AM staining to assess viability and confluence at the cell–electrode interface; stimulated cultures form a dense, confluent monolayer at the electrodes compared with non‐stimulated controls, reproduced from et al. [28], under CC BY 4.0. (k) Intracellular ion flux visualized as time‐lapse fluorescence images of ${\mathrm{Ca}}^{2+}$ in mouse fibroblasts loaded with Fluo‐4FF AM; control cells (no field) and cells exposed to an electric field for up to 10 min show only transient changes and no persistent field‐induced calcium gradient across the cell body (scale bar 20 μm), adapted from Guo et al. [29], under CC BY 4.0.

Dermal fibroblasts possess a resting membrane potential of approximately –70 mV [15, 19]. This potential underpins their responsiveness to external electrical cues, as variations in ionic concentrations across the plasma membrane can profoundly influence cellular behavior. The initiation of a response to an applied electric field primarily arises from the redistribution of intra‐ and extracellular ions, charged proteins, and the activation of voltage‐gated ion channels, as illustrated in Figure 2 [18]. These changes alter the membrane potential and subsequently activate or inhibit multiple signaling pathways, contributing to shifts in proliferation, migration, differentiation, and matrix remodeling [3]. Using these same mechanisms, fibroblasts also respond well to externally applied mechanical cues transmitted through the ECM through mechanosensitive ion channels, integrins, and cytoskeletal linkages. When strain is applied to the skin or a surrogate scaffold, it propagates through the collagen–elastin network, producing local strain fields that regulate fibroblast morphology, proliferation, migration, and matrix synthesis [20, 21].

#### 2.1.1 Ion flux: Electromechanical Sensing, Signaling and Protein Response

Calcium ion flux and membrane‐associated protein redistribution are major drivers of fibroblast responses to electrical stimulation. Bourguignon, Jy, and Bourguignon reported that exposure to high‐voltage pulsed galvanic stimulation rapidly increases calcium influx and insulin‐receptor exposure in human dermal fibroblasts, suggesting polarization of cellular activity through asymmetric receptor clustering [30]. Similar asymmetric redistribution of low‐density lipoprotein receptors was observed by Tank et al., who noted that receptor localization recovered within three hours post‐stimulation (Figure 2a) [22]. While calcium accumulation toward the cathode has been proposed as the main driver of directional migration, Guo et al. demonstrated that calcium ions instead traverse cells through store‐operated calcium channels rather than by cytosolic accumulation [29]. Differences in findings may reflect variations in voltage range and duration; for instance, high‐voltage (>75 V) exposure enhances protein and DNA synthesis [30], whereas lower‐field conditions (5 V/cm) elicit directional migration without sustained calcium retention. Beyond migration and proliferation, electrical fields can also modulate fibroblast differentiation and gene expression. Similarly, hydrostatic or shear stresses on dermal fibroblasts trigger calcium influx through channels such as Piezo1 and TRPV4 [31, 32], and recruit focal adhesion proteins that activate FAK [33], Src, Rho/ROCK [34], TGF‐β [35], and MAPK cascades [1, 34]. Applying 20 mmHg pressure and 0.5 μs shear flow to human dermal fibroblasts, Martyts et al. observed strong calcium responses and increased pYAP/YAP expression consistent with Hippo‐pathway activation [36]. Collectively, these studies underscore the role of calcium flux, either via store‐operated channels or asymmetric accumulation, in modulating fibroblast behavior under electromechanical stimulation, with the magnitude and polarity of the applied electric and strain fields strongly influencing outcomes.

#### 2.1.2 Migration and ECM Remodeling

Electrical and mechanical stimulation not only enhances fibroblast migration, as demonstrated under exposure to a 200 mV/mm electric field by Wang et al. [23] (Figure 2b), but also modulates the intracellular cytoskeletal architecture to facilitate cell motility. This is evidenced by the formation of lamellipodia and the clustering of integrins at the cell edges oriented toward the electric field in stimulated fibroblast cultures (Figure 2c) [24]. This behavior indicates a cause of directional migration that can also modulate the organization of ECM. Xiang et al. demonstrated that microsecond pulsed electric fields induce collagen‐fiber alignment and increase matrix rigidity through enhanced activation of focal‐adhesion proteins such as vinculin and cytoskeletal contraction of filamentous actin (F‐actin) [37]. This process involves elevated focal‐adhesion turnover at 750 V/cm, correlated with enhanced migration, whereas higher intensities (1500 V/cm) stabilised adhesions and reduced motility. Second‐harmonic generation microscopy further confirmed collagen alignment parallel to the electric field, evidencing matrix manipulation mediated by electrical cues. Additionally, Cho et al. reported that exposure to a 20 V/cm alternating current (1–10 Hz) reconfigured actin filaments from aligned cable‐like structures to globular patches [38], likely disrupting network tensegrity and influencing ECM remodeling [21]. Figure 2d highlights the effects of electrical stimulation on cellular reorganization in vivo, resulting in increased interfibrillar spacing and thereby corroborating the in vitro–in vivo effects of electrical stimulation on ECM remodeling [25]. In addition, Figure 2e–h illustrates alterations in the cellular cytoskeleton and focal adhesions, where thicker actin stress fibers and an increased number of focal adhesion sites indicate enhanced cell–ECM tensile interactions, which may underlie the observed global ECM remodeling under the influence of electrical stimulation [26]. The field strength and duration required for directional migration vary widely: while some studies report migration only under high fields (>100 mV/mm) and prolonged exposure (>1 h) [39, 40, 41, 42], others observe electrotaxis at fields as low as 10 mV/mm [43, 44, 45]. This variability suggests that fibroblast migration behavior is less predictable than the intracellular modifications induced by the electric field.

For mechanical stimulation, moderate cyclic strain (20% amplitude at 0.167 Hz for 24 h) was found to promote fibroblast migration (2.26‐fold) [46], aligning cells along the direction of stretch and improving ECM organization. Excessive strain or high‐frequency loading (e.g., 1 Hz) reduce motility (0.44‐fold) [47]. Under biaxial conditions, fibroblasts adopt spindle morphologies, while uniaxial loading aligns both cells and collagen fibers perpendicular to the stretch direction [48]. Shear‐wave stimulation (1–8 kHz) similarly increases fibroblast count, as well as collagen, and elastin content [49]. Collectively, these results confirm that balanced, low‐frequency cyclic strain enhances fibroblast proliferation, migration, and matrix remodeling through YAP/TAZ and FAK‐Src‐Rho/ROCK signaling, whereas over‐stimulation drives myofibroblast differentiation and growth arrest [50, 51].

#### 2.1.3 Proliferation

Fibroblast proliferation has been investigated to assess the influence of both the magnitude and the nature of electromechanical stimulation. Instances of accelerated wound healing in vitro have been reported at applied electric field strengths of 50 and 200 mV/mm in fibroblasts [52] and at 200 mV/mm in keratinocytes [27] as shown in Figure 2i–j. The study by Rouabhia et al. demonstrated a time‐dependent effect of the applied stimulus: cultures exposed to electrical stimulation for 2 h per day did not exhibit a statistically significant increase in the number of viable cells, whereas those stimulated for 4 h per day showed a significant enhancement in cell proliferation after 72 h at both 50 and 200 mV/mm. Furthermore, higher electric field strengths (200 mV/mm) produced significant proliferative effects after 48 h of culture [52]. These observations indicate that the proliferative response is not solely a function of the applied electric field magnitude; rather, the cumulative duration of stimulation also appears to play a critical role in modulating the effect. Foundational work by McLeod, Lee, and Ehrlich systematically examined the effects of electric current, field intensity, and applied frequency on dermal fibroblast cultures [53]. After 12 h of exposure to low‐frequency (1 Hz) and low‐amplitude electric fields (45–90 μV/cm), modulation of the current density revealed a distinct threshold phenomenon. At low current densities (0.1–0.5 μA/${\mathrm{cm}}^{2}$), no significant change in protein synthesis was detected. However, increasing the current density beyond this threshold induced a marked reduction in proline incorporation (used as a proxy for protein synthesis). Notably, within the range of 1–100 μA/${\mathrm{cm}}^{2}$, increasing the current density did not result in further reduction in protein synthesis [53]. When the frequency was varied from 0.1 to 100 Hz at a fixed current density of 1 μA/${\mathrm{cm}}^{2}$, protein synthesis was found to decrease between 1 and 10 Hz, but returned to levels comparable to unstimulated controls as the frequency was further increased into the 100—1000 Hz range [53]. Collectively, these findings indicate that dermal fibroblasts exhibit sensitivity to both the amplitude and the frequency of applied electrical stimulation, implying that modulation of a single parameter is insufficient to reliably control cellular responses. Multi‐axial and dynamic mechanical loading are also commonly employed in fibroblast cultures.A biaxial cyclic stretch at 10% strain and 0.1 Hz was found to increase fibroblast numbers by 75% after three days [54], while equibiaxial loading within collagen hydrogels enhanced collagen deposition and matrix alignment [48]. In contrast, static uniaxial tension beyond 20% or sustained exposure exceeding 192 h reduced proliferation (0.10‐fold) [55]. Dynamic uniaxial loading around 7% strain at 0.0167 Hz induced morphological changes without loss of viability [56].

### 2.2 Keratinocytes

#### 2.2.1 Ion Flux and Membrane Potential

Keratinocytes, the predominant cells of the epidermis, maintain a resting potential of approximately –20 to –30 mV, sustained by voltage‐ and non‐voltage‐dependent ion channels [13]. Gönczi et al. demonstrated that altering ionic composition in the culture medium modulates this potential, thereby influencing keratinocyte morphology and differentiation. When subjected to electrical stimulation, keratinocytes exhibit elevated intracellular calcium levels, confirming activation of voltage‐gated calcium channels, this response is abolished by channel blockers such as verapamil and nifedipine [57]. The response is most pronounced in less‐differentiated cells, suggesting that electrical sensitivity decreases with maturation [57]. Following tissue injury, depolarization and reactivation of bioelectric signaling are observed, indicative of a stress‐response mechanism that promotes migration and re‐epithelialization during wound repair. Keratinocytes are likewise mechanosensitive through chloride and cation channels, integrins, desmosomes, and cytoskeletal networks that transduce strain into biochemical signaling. Stretch‐activated channels and integrin‐based adhesions trigger calcium influx and downstream activation of FAK–Src [58], Rho/ROCK, TGF‐β, Hippo/YAP/TAZ, MAPK [59, 60], and Wnt pathways [1]. These cascades coordinate cytoskeletal dynamics and transcriptional responses governing proliferation and differentiation.

#### 2.2.2 Migration and Barrier Formation

Keratinocytes are particularly susceptible to directional migration (galvanotaxis), typically moving toward the cathode in vitro, which is analogous to the endogenous electric cues guiding wound closure [61]. In custom‐designed bioreactors, modulation of the electric‐field direction enables programmable control of keratinocyte migration [62]. Zajdel et al. demonstrated precise, reversible steering of primary keratinocytes under a 1—2 V/cm field, with migration toward the cathode within 90 min [62]. This capacity for guided migration highlights the utility of electrical stimulation for epithelial patterning and wound‐closure applications. Migration arises from the activation of voltage‐gated ion channels and asymmetric redistribution of charged proteins such as epidermal growth‐factor receptors, integrins, and cadherins, that trigger intracellular cascades including MAPK/ERK [63, 64], PI3K/Akt [9], and PLCγ, culminating in cytoskeletal rearrangement and directed motility. Electrical cues can also influence keratinocyte differentiation [65, 66] and protein expression. Mechanical inputs regulate keratinocyte‐derived ECM by altering deposition of collagen, laminin, and fibronectin [67, 68]. Low‐frequency shear or vibration enhances ECM density and alignment, supporting barrier restoration, while overstimulation disrupts adhesion junctions and promotes terminal differentiation.

Similarly, keratinocyte migration and re‐epithelialization are enhanced under mild cyclic strain and low‐frequency vibration. Small displacements (0.4 μm) or accelerations (1.2$\times 10^{-5}$ m $s^{-2}$) increased migration and proliferation (1.8‐fold) [69], whereas excessive frequency (80 Hz) or high strain reduced migration (0.44‐fold) [69]. Cyclic stretch promotes lamellipodia formation and directional movement, while static compression can induce differentiation. Through YAP/TAZ activation, mechanical stimuli modulate keratin gene expression and barrier formation, linking external strain directly to epidermal maintenance.

#### 2.2.3 Proliferation

Systematic evaluation of keratinocyte proliferation under electrical stimulation remains less well‐characterized than migration, potentially due to the direct role played by keratinocyte motility in initiatiating wound closure. Several studies demonstrate the proliferative potential of applying in vitro electrical stimulation to keratinocyte cultures. Investigations by Rouabhia et al. assessed the effect of stimulation durations ranging from 6 to 24 h under an applied electric field of 50—200 mV/mm [63]. The initial analysis highlighted a clear interaction between exposure time and electric field strength: maximal cell proliferation was observed at 100 mV/mm following 24 h of stimulation, whereas under the 6 h regime the highest optical density was recorded at 200 mV/mm, with a progressive increase in relative cell number from 50 to 200 mV/mm. This pattern contrasts with the reduced proliferative response observed at field strengths above 100 mV/mm in the 24 h group [63]. Considering proliferative outcomes alone, these findings indicate that stimulation duration and electric field magnitude are interdependent parameters that jointly determine the biological response. Similar findings reported by Li et al. corroborate these observations, demonstrating that increasing the stimulation pulse duration while applying 5.4 V (54 mV/mm) at 60 Hz for 6 h resulted in a 1.05‐fold increase in cell viability at 1 μs and a 1.13‐fold increase at 1000 μs, relative to non‐stimulated controls [44]. Although the absolute enhancement in proliferative behavior was modest, these results further underscore the existence of a time‐dependent stimulation effect.

Keratinocyte proliferation responds optimally to moderate mechanical loading. A 10% strain at 0.1 Hz increased cell number by roughly 75% in dynamic collagen gel systems [54]. Strain amplitude of 10% combined with 24 h exposure yielded a 2.39‐fold increase [59], whereas higher strain (20%) or very long exposure (96 h) suppressed proliferation (0.66‐fold) [67]. Shear and compression modes also upregulated ECM components, while vibration‐induced compressive waves at 75 Hz increased collagen, elastin, and fibrillin protein expression [70]. The collective evidence indicates that keratinocyte proliferation favors mid‐range cyclic strain (5—15%), low frequency (1 Hz), and short‐to‐intermediate stimulation periods (<24 h).

#### 2.2.4 Key Insights

In both cell types, these integrated findings illustrate that moderate, cyclic mechanical stimulation optimally promotes proliferation, migration, and ECM organization. Such insights guide the rational design of next‐generation bioreactors for in vitro skin mechanobiology and regenerative medicine applications. In the context of electrical stimulation, the current understanding is less definitive. The heterogeneous cellular responses observed under similar stimulation parameters necessitate a more detailed analysis of the available data to identify robust and biologically meaningful trends rater than extraction from a pre‐existing unified narrative review.

## 3 In Vitro Strategies and Setups

### 3.1 Electrical Stimulation

**FIGURE 3:**
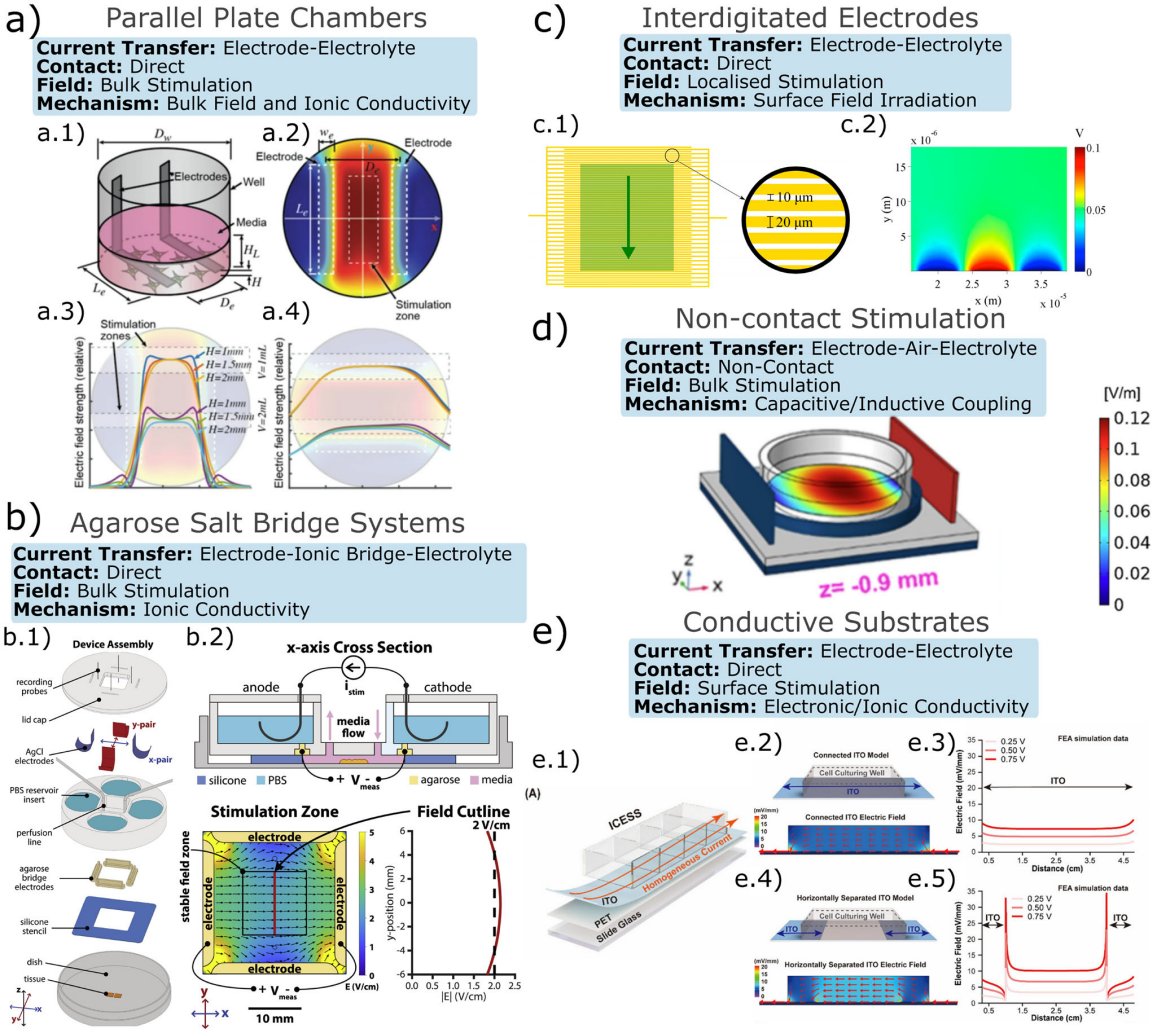
Representative electrical stimulation set‐ups used to generate controlled electric fields in vitro. (a.1) Parallel plate chambers showing the design of a well containing vertically oriented electrodes and culture medium. (a.2) Finite element simulations of the relative electric field distribution across the well bottom. (a.3) Line profiles along orthogonal axes through the center of the well illustrate how field strength varies with medium height and electrode immersion depth, reproduced from Cibrão et al. [71], under CC BY‐NC‐ND 4.0. (b) Agarose salt bridge systems, (b.1) highlighting the SCHEEPDOG device assembly in a Petri dish, in which a silicone stencil defines the central stimulation zone and four agarose bridges connect Ag/AgCl electrodes in a PBS reservoir to the culture region. (b.2) A sectional schematic shows the anode/cathode pair and media flow, alongside numerical simulations indicating a stable, uniform field zone between the opposing agarose electrodes, reproduced from Zajdel et al. [62], under CC BY 4.0. (c) Interdigitated electrodes. (c.1) Plan view of a gold interdigitated electrode (IDE) structure used to generate highly non‐uniform electric fields at the cell–substrate interface, with a magnified region highlighting the digit width and spacing. c.2) Finite element simulations show the resulting electric potential distribution around IDE fingers, reproduced from MacKay et al. [72], under CC BY 4.0. (d) Non‐contact stimulation. Contactless configuration in which external plate electrodes placed on either side of a plastic culture dish induce an electric field across the cell layer; the color map shows the spatial distribution of field magnitude on a horizontal plane, reproduced from Zironi et al. [73], under CC BY‐NC‐ND 4.0. (e) Conductive substrates. (e.1) Indium–tin‐oxide (ITO)–based integrated conductive electrical stimulation system in which an ITO layer on the underside of a culture well functions either as a laterally connected electrode (e.2–3) or as horizontally separated ITO track (e.4‐5). Finite element simulations of one‐dimensional electric field profiles along the culture surface for different applied voltages (0.25–0.75 V), reproduced from Rahaman et al. [28], under CC BY 4.0.

A wide range of stimulation set‐ups have been created to investigate the effect of electrical stimulation, in vitro the most common of which are presented in Figure 3. Each configuration comprises the following fundamental components: (1) a power supply, (2) electrodes, (3) a stimulation culture chamber, and (4) an incubator. Electrodes serve as the critical interface between the stimulation apparatus and the cell culture. Selection of the electrode material is a pivotal consideration in the design of an electrical stimulation system to ensure biocompatibility and to mitigate unwanted electrochemical reactions at the interface. Furthermore, in conjunction with the strategic placement of electrodes, material selection is instrumental in defining the electric field encountered within the culture system. Power supplies determine the programmable output parameters, including voltage, current, frequency, duty cycle, and waveform (see Table 1). Although standard power sources facilitate preprogrammed control over the administered stimulation parameters, there is a growing implementation of control loop systems. These systems incorporate microcontrollers capable of both sensing and stimulation, operating in accordance with pre‐established thresholds that enable cellular stimulation in response to specific stimuli [74].

**TABLE 1:** Typical electrical and mechanical stimulation parameters extracted from the literature and the units used for data analysis.

Stimulation parameter	DC/AC/Both	Description	Units
**Applied Voltage**	Both	Applied potential difference between electrodes	V
**Applied Current**	Both	Flow of current between electrodes	A
**Electric Field Strength**	Both	Electric field imposed through the stimulation set up	mV/mm
**Electrode Separation Distance**	Both	Distance between electrodes	mm
**Bias**	AC	the voltage offset for the AC electrical signal to determine an effective 'zero' point. This is > 0 V for a positive bias, < 0 V for a negative bias and 0 V when no bias is applied.	V
**Electrode Material**	Both		—
**Applied Pressure**	—	Pressure used to stimulate cells, often induced by a pneumatic physical load.	N/${\mathrm{mm}}^{2}$
**Strain Amplitude**	—	Maximum strain acheived per stimulation cycle.	%
**Strain Rate**	—	Rate of change in the applied strain.	%/s
**Shear Stress**	—	Forces acting parallel to the culture surface.	N/${\mathrm{mm}}^{2}$
**Substrate Stiffness**	—	Young's modulus of the culture platform.	N/${\mathrm{mm}}^{2}$
**Waveform**	Both	The cycle shape of the signal e.g. sine, square or pulsed.	—
**Frequency of waveform cycle**	Both	the time to complete a single waveform.	Hz
**Duty Cycle**	Both	The active period of the stimulation waveform.	%
**Application Frequency**	Both	Repeated occurrence of the electrical stimulation procedure	hrs, days
**Duration**	Both	Total stimulation time	min, hrs, days, weeks

#### 3.1.1 Parallel Plate Arrays

The most prevalent and straightforward configuration is the parallel plate array (Figure 3a1), typically comprising two parallel plate electrodes. These electrodes are generally fabricated from precious metals such as platinum or silver, or alternatively constructed by coating non‐conductive substrates with a conductive particulate layer such as carbon. The electrodes remain submerged throughout the stimulation process and can be subsequently removed to facilitate other experimental procedures, or mitigate potential cytotoxic effects from prolonged exposure and oxidative residue leaching into the culture media. During stimulation, a potential difference is applied between the plates, resulting in a predominantly uniform electric field across the culture dish (Figure 3a2) [71]. As illustrated in Figure 3a3–4, the electric field exhibits a relatively consistent distribution across the central plane, with pronounced reductions near the media‐electrode interfaces [71]. In contrast to more complex systems, these configurations are compatible with standard cell culture dishes and can be produced efficiently on a large scale at minimal cost. However, the direct interfacing of electrodes with the culture can result in cell death due to electrochemical by‐products arising from electrolysis of the medium, which generates free radicals and metal ions, thereby damaging the cells and potentially altering the pH of the medium. Additionally, redox reactions occurring at the electrode–electrolyte interface generate gas bubbles whose subsequent collapse can induce localized mechanical stress, thereby disrupting cell membrane integrity and compromising cell–substrate adhesion sites, ultimately resulting in cellular damage or detachment. The limited spatial control inherent in this setup restricts stimulation to the bulk culture, offering poor resolution at the single‐cell level. Furthermore, at elevated voltages, Joule heating may occur as a consequence of the current passing through a highly resistive medium.

#### 3.1.2 Agarose Salt Bridges

Agarose salt bridge electrodes are frequently employed as non‐leaching alternatives bridging conductive metals and the cell culture medium. A thin layer of agarose is used to transmit the electrical signal from electrodes, which are typically composed of Ag/AgCl (Figure 3b) [62]. This allows stimulation without direct contact between the cells and electrodes, thereby eliminating the risk of electrode degradation and contamination of the medium from electrolytic reactions. Moreover, the stability of stimulation is enhanced due to minimal pH effects on the medium. Dermal fibroblast migration studies by Snyder, DeJulius, and Willits and keratinocyte migratory responses explored by Zajdel et al. represent two key examples using agar salt bridges as effective biocompatible electrodes for electric field generation [40, 62]. However, these highly resistive gels may necessitate the application of higher voltages to compensate for reduced charge transfer to the surrounding medium. Furthermore, variability in gel composition and thickness complicates system assembly, both because of the challenges associated with fabricating and isolating salt bridges and because the resulting variability in electrical resistance often requires experiment‐specific adjustment of stimulation parameters to achieve comparable power outputs across trials.

#### 3.1.3 Interdigitated Electrodes

Interdigitated electrodes enhance spatial control over electric fields through interlocking surface‐patterned microelectrodes, typically composed of gold, platinum, or conductive substrates [72, 75]. Application of a voltage across the alternating fingers of the electrode results in a non‐uniform electric field between the gaps, which arcs between the adjacent electrode surfaces. By producing irradiating fields from the electrode surfaces, the arcs facilitate spatial control of the electric fields not only between the conductive substrates but also within the culture medium (Figure 3c1‐2) [72]. In contrast to many removable or bulk stimulation systems, interdigitated electrodes offer a level of spatial control for electrical stimulation that is beneficial for targeted stimulation and highly sensitive impedance measurements. However, like all direct contact methods, there are inherent risks associated with material cytotoxicity. Moreover, the fabrication of microelectrode arrays requires the use of high‐precision instrumentation to achieve high‐resolution manufacturing, which can lead to spatially non‐uniform electric fields and thereby hinder the standardization and reproducibility of stimulation studies.

#### 3.1.4 Non‐Contact Stimulation

Non‐contact stimulation represents an indirect approach by positioning the electrodes on either side of the culture plate and applying a potential difference (Figure 3d) [73, 76]. The electric field is insulated by the material of the culture plate, which is typically plastic or glass, thus preventing any current from flowing through the medium. Such configurations mitigate the risk of cytotoxic by‐products associated with direct electrode contact. The magnitude of the electric field is significantly influenced by the distance between the electrodes, with a pronounced decrease in the effective field observed as separation increases [73, 76]. This phenomenon is attributable to the high impedance provided by both the air and the insulating culture plate. To counteract this attenuation, a higher voltage must be applied, which often necessitates more powerful (and thus expensive) power supplies and rigorous safety protocols. Similar to parallel plate electrode arrays, these systems are also limited to bulk stimulation with minimal spatial control.

#### 3.1.5 Conductive Substrates

Conductive substrates are typically thin conductive films that coat culture surfaces to establish an electric field across the material. The field irradiates from the surface to the attached cells or between distinct surface‐coated electrodes, as illustrated in Figure 3e. This configuration provides a direct electrical interface between the entire culture and the electrodes, which are often fabricated from a thin, transparent layer of conductive polymer, such as Poly(3,4‐ethylenedioxythiophene) polystyrene sulfonate (PEDOT:PSS), indium tin oxide (ITO), or commonly Polypyrrole (PPy) doped Poly‐L‐lactic acid (PLLA) films, often cast onto culture plates or glass slides [52, 63, 77, 78, 79, 80, 81]. When applied as a homogeneous layer on the culture plate, the electric field aligns parallel to the plate, exhibiting minimal deviation in field strength across the structure. This phenomenon arises because the field is generated by the potential difference between the conductive surface, flowing with charge due to an applied potential, and the surrounding medium, as depicted in Figure 3e2–3 [28]. Modifying the surface configuration from a single electrode layer to a two‐electrode setup also alters the imposed electric field. This effect is noted in Figure 3e4–5, where a pronounced spike in the electric field is observed at the edge of the electrode, while remaining relatively constant through the central region of the plate, since the electric field is contingent on the potential difference between the electrodes instead of merely the surrounding medium. The primary advantage of these configurations is their optically transparent surface, which allows for a variety of data extraction methods ranging from standard viability and cell population counts to detailed live imaging of cultures during stimulation [28]. The direct contact between the culture surface and the electrode ensures that the applied electric field stimulation can be controlled and characterized. However, the dimensionality of this set‐up limits experimental parameters, rendering it unsuitable for testing 3D or co‐culture models. Furthermore, redox reactions at the electrode surface that exceed the electrolytic potential of the medium (1.23 V for pure water) can result in bubble formation, potentially detaching cells adhered to the surface, and therefore constraining the applied fields and currents. Furthermore, beyond a certain voltage threshold, electrochemical reactions trigger the overproduction of Reactive Oxygen Species (ROS) and free radicals. The concentration of such species can determine the ultimate cellular response; low doses have been found to promote pathways such as MAPK/ERK or PI3K/Akt for (over)growth, whereas high doses can be inhibitory through oxidative damage of lipids and proteins, dampening the regenerative response [82, 83].

### 3.2 Mechanical Stimulation

Within the field of tissue engineering, it is well recognised that the application of exogenous mechanical loading can be harnessed to regulate cellular activity [21, 90]. This principle underpins many bioreactor systems designed to enhance tissue yield, as well as regenerative medicine strategies that aim to expedite tissue repair in contexts such as wound healing. The type of strain experienced by cells within the ECM is frequently biaxial, with multiple strain modes depending on tissue location and physiological function. For instance, the skin can simultaneously undergo compression, stretching, and shearing during movement. Consequently, to determine which strain modalities most effectively induce cellular responses, researchers have developed a range of loading platforms for in vitro cell culture. This section examines existing mechanical stimulation configurations and the spectrum of applied parameters, providing a comprehensive overview of the principal mechanical cues that modulate cellular behavior.

#### 3.2.1 Tensile Loading

**FIGURE 4:**
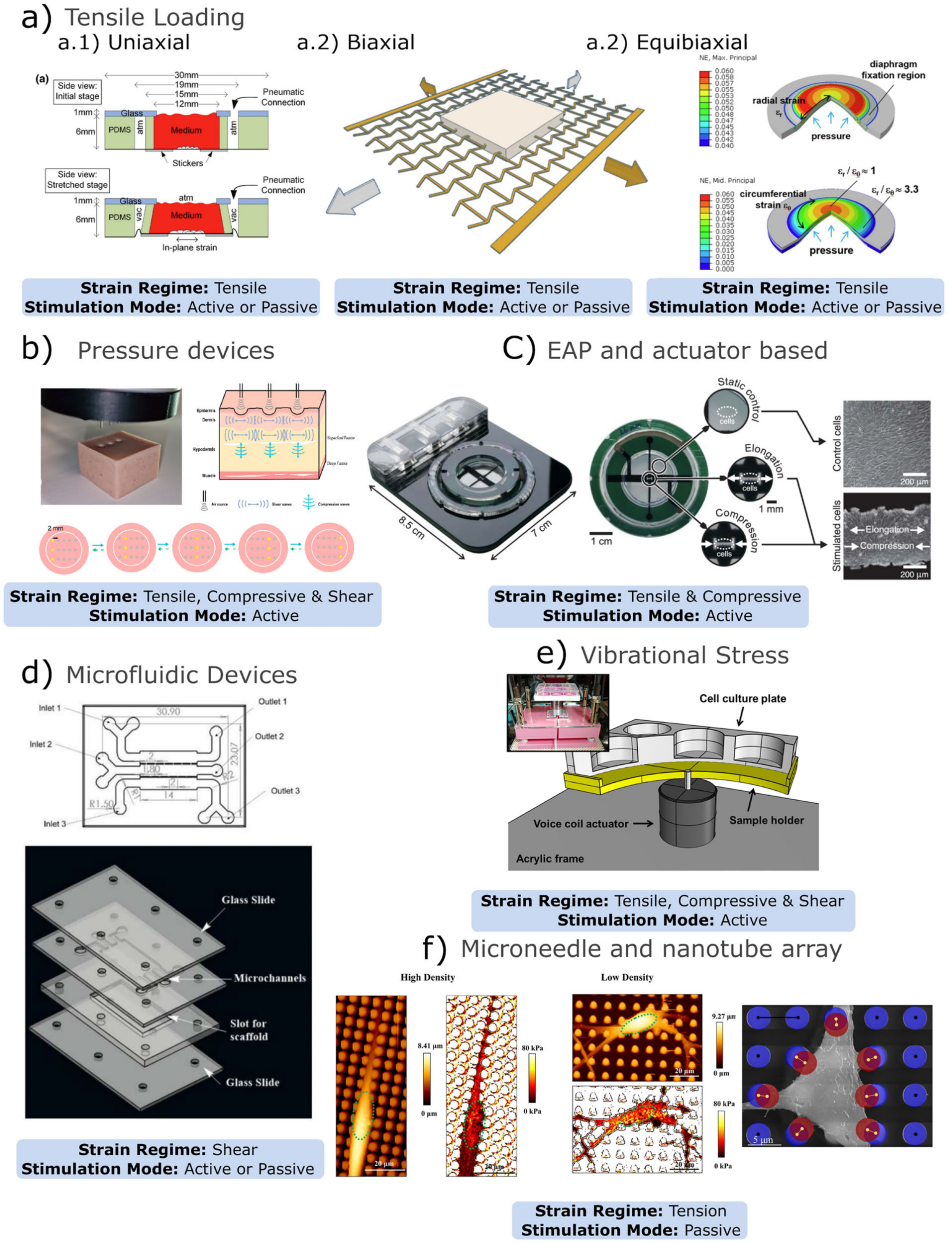
Mechanical stimulation set‐ups used for in vitro loading of skin‐relevant cells, illustrating tensile loading, pressure devices, electroactive polymer–based (EAP) actuators, vibrational stress, microfluidic platforms and micropillar arrays. (a.1) Tensile loading devices: side‐view schematic of a pneumatically driven stretching system in its initial and stretched states, in which a PDMS membrane carrying the cells is clamped between glass plates and elongated in‐plane, reproduced from Kreutzer et al. [84], under CC BY 4.0. (a.2) Biaxial straining device in which an auxetic mesh is embedded in a tissue culture substrate; an imposed strain in one direction (yellow arrow) produces a coupled lateral expansion of the mesh and underlying membrane (grey arrow), reproduced from Wei et al. [85], under CC BY 4.0. (a.3) Pressure‐driven diaphragm bioreactor showing the finite element predictions of hydrostatically induced radial and circumferential strains on PDMS diaphragms, reproduced from Maghin et al. [86], under CC BY 4.0. (b) Pressure‐based surface wave/indentation system for skin stimulation: practical implementation on silicone, schematic of the stimulus applied to skin, and layout of three air outlets mounted on a robotic arm that sweep along the Y‐axis to generate traveling pressure waves across reconstructed skin, reproduced from Qiao et al. [49], under CC BY 4.0. (c) Electroactive polymer and actuator‐based stretcher: dielectric elastomer actuator (DEA) device mounted in a compact holder, with patterned electrodes surrounding a central culture region so that actuation generates uniaxial tensile or compressive strain; the device can reproduce complex physiological strain–time profiles, reproduced from Poulin et al. [87], under CC BY 4.0. (d) Microfluidic device with three inlets and outlets to deliver media, trypsin and shear flow to create a wound region and 3D/exploded views illustrate the complete chip assembly, reproduced from Gupta et al. [88], under CC BY 4.0. (e) Vibrational stress culture model consisting of an acrylic frame, voice coil actuator and sample holder located inside the incubator, to apply controlled oscillatory loading to standard culture plates, reproduced from Kim and Kwon [60], under CC BY 4.0. (f) Deflection maps of cell‐induced bending of high‐ and low‐density micropillar arrays used to quantify local traction forces. Distributions of cell morphology and apparent stiffness on high‐ versus low‐density micropillar arrays, showing how pillar spacing modulates cytoplasmic organisation and mechanical properties, reproduced from Feng et al. [89], under CC BY 4.0.

Uniaxial [84], biaxial [85] and equibiaxial [86] tensile loading systems (Figure 4a1‐3, respectively) apply elongation to clamped, cell‐seeded substrates such as hydrogels, lyophilized scaffolds, or flexible elastomeric membranes. The primary difference lies in the strain field. Biaxial systems generate a more homogeneous field, ensuring that each cell experiences similar deformation [85], whereas uniaxial designs produce gradients where strain decreases laterally from the central axis [84]. These systems are implemented through mechanical frames, vacuum‐driven membranes, or inflatable chambers that deliver cyclic or static stretch under controlled frequency, amplitude, and duration.

#### 3.2.2 Uniaxial Strain

Uniaxial systems frequently display strain gradients that limit homogeneity across the sample, making results dependent on the maximum strain region. Moreover, these gradients introduce substantial heterogeneity in the overall strain field of the specimen under stimulation, affecting both the near‐field and far‐field deformation regimes. Typical devices comprise a fixed‐grip frame or modified culture plate attached to a cyclic stretching motor. Examples include static elongations (0%–40% can be applied to engineered skin constructs), where collagen alignment increased monotonically with strain but tissue viability and strength peaked at intermediate strain [91]. A custom cyclic stretching unit operating at 7% cyclic strain at 0.0167 Hz by Xie et al. revealed that their polyurethane‐membrane substrate exhibited a non‐uniform strain field, illustrating the challenges of uniaxial platforms. Furthermore, a spring‐based uniaxial tension device applied to explanted human burns produced marked tissue contraction over six days [50]. These examples show that uniaxial strain bioreactors effectively mimic directional mechanical cues but inherently generate heterogeneous deformation across the culture surface. Additionally, microneedle and nanotube arrays (Figure 4f) can be employed to impose tensile strains on adherent cells by modulating the spacing and number of pillars within the array. This geometric tuning alters the density and distribution of available adhesion sites, thereby regulating the efficiency of strain transmission from individual micropillars to single cells. Thus, geometric tuning can effectively enable the application of dynamic mechanical strain on otherwise static substrates [89].

#### 3.2.3 Biaxial Strain

Biaxial strain devices, including vacuum‐inflated membranes and inflatable collagen gels, provide more uniform deformation. Such systems enable controlled equibiaxial stretching and more reproducible analysis of mechanotransductive responses [48, 54].

#### 3.2.4 Shear Strain

Shear strain devices apply tangential forces at cell–substrate or cell–fluid interfaces. They include rotating‐cone viscometers, parallel‐plate microfluidic flow chambers (Figure 4d), and emerging contact‐free modalities (Figure 4b,e) [49, 60]. Furthermore, systems such as air‐driven shear‐wave stimulation (SWS) by Qiao et al. generate shear and compression waves through the culture medium [49], enabling high‐frequency shear excitation (1 Hz–8 kHz) without direct contact, providing an alternative to traditional fluid‐flow or compression bioreactors (Figure 4b).

#### 3.2.5 Compression

Compression bioreactors impose normal forces perpendicular to the cell layer using piston‐driven plungers, membrane inflation, or vibrational platforms. Vibrational devices apply oscillatory compressive strain across frequencies from 40 Hz to 120 Hz, while ultrasound systems generate high‐frequency pressure waves. One example includes, the torque‐based vibration device employed by Caberlotto et al., capable of 40–120 Hz cyclic compression to probe skin mechanobiology [92]; related in vivo studies at 75 Hz showed enhanced ECM protein expression, including collagen, elastin, and fibrillin [70]. Unlike tensile loading, which primarily elongates constructs, compressive loading can drive local buckling or out‐of‐plane deformation of soft tissues and hydrogels, leading to non‐uniform strain fields and potential stress concentrations. Such buckling phenomena are particularly relevant when comparing compression versus tension experiments, as they can alter cell morphology, fluid transport, and mechanotransduction pathways, and therefore should be considered when interpreting bioreactor‐induced responses. Recent advances in smart materials, particularly the integration of dielectric elastomer membranes into cell culture systems, have enabled the development of devices capable of reproducing complex tissue‐like motion on a two‐dimensional, mechanically tunable platform (Figure 4c) [87]. This provides a controllable in vitro environment in which physiologically realistic mechanical cues can be systematically applied to cells, thereby bridging the gap between traditional static culture systems and the dynamic mechanical conditions experienced in native tissues [87].

### 3.3 Electromechanical Stimulation

Beyond isolated stimulation modalities, the field is increasingly progressing toward hybrid electromechanical platforms that integrate mechanical and electrical cues within a single system. These approaches typically employ smart functional materials, such as piezoelectric [93, 94, 95] and triboelectric [96, 97, 98] polymers, which act as dual‐mode actuators by transducing mechanical deformation directly into electrical current. Although such devices have demonstrated considerable potential in cardiovascular [99, 100] and peripheral nerve repair [95, 101], their application to topical wound‐healing dressings remains at a relatively early stage [93, 96, 97, 102].

Within these integrated systems, cellular behavior is governed by the interplay between mechanical forces and electric fields. However, because the resultant stimulation profiles are strongly contingent on specific device architectures and material configurations (e.g., fiber orientation, scaffold geometry, and composite layering), current hybrid studies do not provide sufficiently standardized input parameters to enable the type of comparative, quantitative analysis undertaken in this review. Accordingly, the present study restricts its scope to isolated stimulation paradigms, with the aim of more rigorously defining the fundamental dose–response relationships that will underpin the rational design and optimization of future hybrid electromechanical platforms.

### 3.4 Standardizing Across Experiments

Effective electrical and mechanical stimulation systems for in vitro studies need precise design and characterization of stimulation parameters. Balancing advantages and limitations of available setups is crucial to meet experimental needs. Exploring various electrode configurations, like parallel plate arrays and interlocking microelectrodes, underscores the challenges of precise electric field control and risk mitigation from cytotoxicity and electrochemical interference. Similarly, while both uniaxial and biaxial systems have become standard tools for modeling skin tension in vitro, the methodological diversity and differences in the effective strain and stress fields experienced by cells continue to limit cross‐study comparability. To address these discrepancies, advances in device design and materials to engineer the desired strain and electric fields aim to improve scalability and safety, supporting detailed and reproducible research. Innovation is vital to overcome challenges like spatial accuracy and adaptability in experimental and clinical contexts.

While this study has standardized key experimental parameters with respect to the biological outputs for cross‐study comparison, it must be noted that significant variability in exists in experimental design when using custom‐made stimulation bioreactors. These include variations in electrode materials, and thus the variations in waveform characteristics, substrate compliance, electrode conductivity, the inherent bioactivity of the substrates, as well as the biocompatibility of interfacing materials in the bioreactors. In this study, the aggregated trends from the literature do not account for the standardization of these parameters, primarily due to the limited or incomplete reporting of these elements. Thus, the potential of confounding variables must not be ignored, underscoring the need for standardized reporting of systems for cell culture assays, as previously proposed by Sakurai et al. [103].

## 4 Data‐Driven Insights on Exogenous Fibroblast and Keratinocyte Stimulation

The data‐driven analysis consolidates over 390 individual experimental data points for electrical stimulation and 170 for mechanical stimulation extracted from the literature pertaining to the respective modalities of skin cell stimulation. The datasets span studies on both fibroblasts and keratinocytes, representing a range of in vitro experimental setups, culture formats (including 2D monolayers, 3D hydrogels and scaffolds, or microfluidic chambers), and species origin (such as human, murine, and porcine). For electrical stimulation, the set ups are primarily distinguished by different electrode geometries, whereas for mechanical stimulation these are expressed through variable mechanical boundary conditions. This section integrates the results of the parameter‐based visualization analysis, highlighting single‐parameter dependencies, pairwise interactions, and combined electrical metrics that modulate proliferation and migration responses for each cell type. These stimulation conditions were standardized by normalizing key exposure parameters, such as field strength, applied current, stimulation frequency, and related waveform characteristics, into a common set of quantitative metrics. This standardization allowed direct comparison between studies and experimental setups.

### 4.1 Survival and Proliferation ‐ Single Parameter Trends

### 4.2 Fibroblasts

**FIGURE 5:**
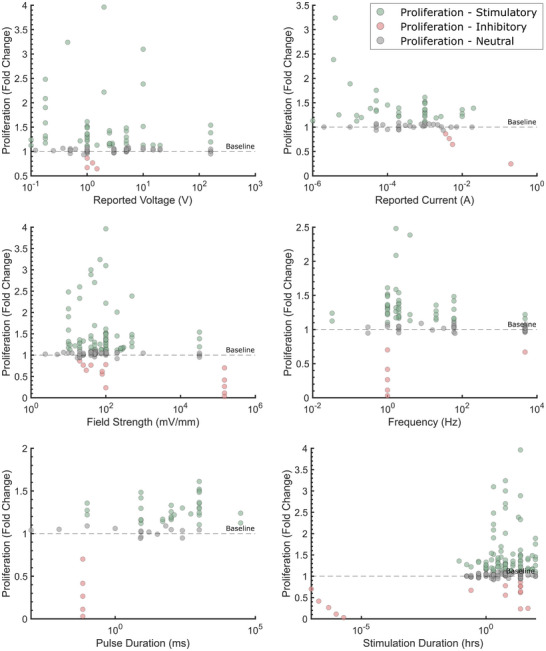
Global parameter analysis of electrical stimulation effects on fibroblast proliferation. Comprehensive visualization of normalized proliferative outcomes plotted against key electrical stimulation parameters, including reported voltage, current, field strength, frequency, and pulse duration. Data points are color‐coded to visualize the efficacy of stimulation: Green indicates a stimulatory proliferative response (>1.1‐fold change), Red indicates inhibition (<0.9‐fold change), and Grey indicates a neutral outcome. The dashed line (y=1.0) denotes the control baseline. Parameters with wide variance are plotted on a logarithmic x‐axis to facilitate trend visualization. Data points were compiled from literature sources listed in the supplementary material.

Figure 5 illustrates the effects of a individual parameters on cellular proliferation. The voltage range examined spans from 0.1 to 160 V, with a notable aggregation of data around lower to moderate voltages (<20 V) where an enhancement in proliferation is predominantly observed. The maximum proliferative response reported in the analyzed literature corresponded to a 3.96‐fold increase relative to the control growth rate when an electrical potential of 2 V was applied to the culture [81]. In contrast, inhibitory effects were observed at voltages between 1 and 1.5 V, with a 0.65‐fold response [28]. Across the entire voltage spectrum, a baseline proliferative response of 1.00‐fold was documented, indicating no change in proliferation. However, no distinct trend emerged, possibly attributable to the limited reporting of inhibitory effects in the existing literature.

A similar pattern was observed in the context of electric field strength. The majority of documented instances are concentrated within the wound electric field range, extending up to 500 mV/mm, with only a few reports of stronger fields. An optimum fold change in proliferation was recorded at 100 mV/mm, whereby fibroblasts achieved a peak proliferation rate of 3.96 times higher than its control [81]. Beyond this level, extremely high fields, such as 1.5$\times 10^{5}$ mV/mm, were associated with a marked decrease in proliferation to 0.03‐fold [104] or a partial suppression to a 0.70‐fold rate of growth [104]. Conversely, at the lower end of the spectrum, weak fields in the range of 2.4 mV/mm resulted in only a marginal stimulation of proliferation, evidenced by a 1.02‐fold increase [28]. Given the direct correlation between voltage, current, and field strength, these results suggest that the intensity of the local electric field within the culture is not necessarily the predominant factor influencing the proliferative response.

Current intensity demonstrates the most distinct monotonic trend. The peak stimulation, a 3.24‐fold increase, was observed at a current of 4 × 10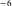 A [105]. In contrast, currents exceeding 0.001 A resulted in a rapid decline in proliferation, culminating in a 0.25‐fold reduction at 0.2 A [106]. Currents within the intermediate range of 1 × 10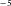 to 1 × 10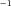 A yielded results that were largely neutral. However, currents in the microampere range, particularly around 1 × 10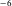 A, exhibited a heightened incidence of stimulatory effects. These findings imply that it is the excessive transfer of charge and the resultant electrochemical alterations, rather than voltage, that may impede fibroblast proliferation.

Electrically, the applied frequency, pulse duration, and stimulation time exhibited variability, yet discernible limits were apparent. Cellular proliferation reached its apex at approximately 1.69 Hz, with an observed increase of 2.48‐fold [43]. In contrast, stimulation at 1 Hz elicited both an enhancement in cellular response, with a 1.16‐fold increase, and a suppression to 0.03‐fold [104]. Pulses with a duration of 1000 ms resulted in a moderate amplification of 1.61‐fold [107], whereas ultrashort pulses of 0.07 ms were significantly inhibitory, reducing response to 0.03‐fold [104]. The duration of exposure adhered to a similar pattern: a 24‐hour exposure achieved the maximum proliferation, observed at 3.96‐fold [81], while an almost instantaneous exposure of 1.94$\times 10^{-6}$ h nearly eradicated growth, resulting in a 0.03‐fold change [104]. Treatments extended to 96 hours showed partial normalization of the response, with a 1.25‐fold increase [77]. Across the various ranges of each parameter, neutral outcomes, indicating a 1.00‐fold change, were also reported.

**FIGURE 6:**
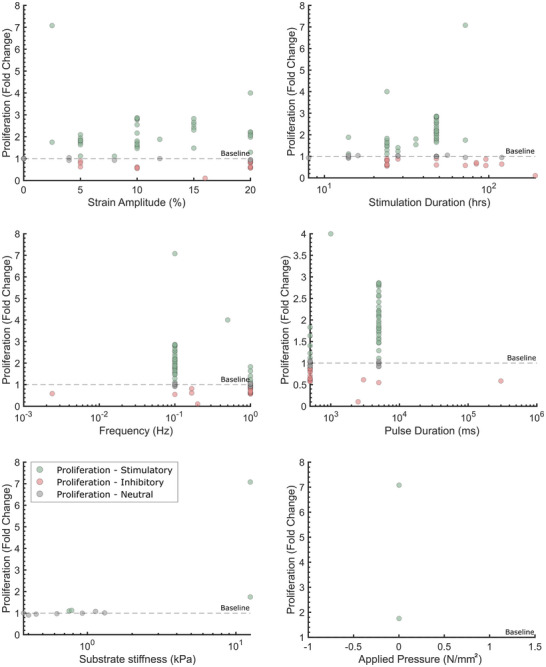
Global analysis of mechanical stimulation parameters on fibroblast proliferation. Aggregated literature data displaying the normalized proliferative outcome (fold change relative to unstimulated controls) as a function of various mechanical parameters, including displacement, strain amplitude, frequency, and substrate stiffness. Data points represent individual experimental outcomes. colors denote the nature of the response based on a defined threshold: Green circles indicate a stimulatory effect (>1.1‐fold change), Red circles indicate an inhibitory effect (<0.9‐fold change), and Grey circles indicate a neutral response (0.9–1.1‐fold change). The dashed horizontal line at y=1.0 represents the baseline control value. x‐axes are presented on a logarithmic scale where data ranges span multiple orders of magnitude. Data points were compiled from literature sources listed in the supplementary material.

As seen in Figure 6, mechanical stimulation studies report a compact but informative landscape for fibroblast proliferation, where amplitude, substrate mechanics and exposure time interact to produce either very large stimulation or near‐complete suppression. Strain amplitude and substrate stiffness in particular identify an optimal mechanical window: maximal proliferation (7.08‐fold) was observed with modest strain and moderate stiffness conditions (strain amplitude 2.5%, substrate stiffness 12.5 kPa [54], whereas large amplitudes and prolonged exposure produced strong inhibition (0.10‐fold at strain amplitude 16 and 192 h stimulation [55]. The same experimental set shows frequency sensitivity: low cyclical drives (0.1 Hz) coincide with the largest reported stimulation (7.08 [54]), whereas slightly higher cyclical rates (0.2 Hz) were associated with severe suppression (0.10 [55]). However it is important to recognize the inhibitory effects across the strain amplitude and frequency ranges which may suggest that other factors limit stimulatory effects for these otherwise favorable conditions.

Pulse timing and waveform of mechanical stimulation further refine this window. Pulse widths of 1000 ms produced appreciable stimulation (4.00‐fold) [108], while longer pulses around 2500 ms were linked with the strongest inhibition (0.10‐fold [55]); mid‐range pulses and durations clustered near neutrality (e.g. 5000 ms pulse, 48 h exposure 1.00 [109]).

The reported literature collectively identifies an optimal range characterized by low voltage applications (1–2 V), microampere‐scale currents, and field strengths approximating 100 mV/mm. Deviations towards increased electrical load or extended exposure durations have been observed to induce a shift in fibroblast activity from stimulatory to inhibitory; however, this phenomenon is not uniformly reported across all studies. Consequently, fibroblast proliferation is contingent upon a harmonious electrochemical milieu rather than being dictated by any singular parameter. Mechanically, fibroblast proliferation is sensitive to the change in substrate stiffness (appearing to be optimum at 12.5 kPa in the compiled data), cyclic strain, low frequency (0.1–1 Hz), intermediate pulse widths, and intermediate exposure durations (tens of hours) increases in strain amplitude, pulse width or prolonged stimulation may push the response toward inhibition of replication or low survival [54, 55, 108, 109]. Yet, due to the lack of strain amplitudes outside of the 0%–20% range and limited variation of frequency and stimulation duration applied to fibroblast cultures, minimal conclusions can be drawn regarding the optimal strain amplitudes and frequencies from the compiled literature.

#### 4.2.1 Migration ‐ Single Parameter Trends

**FIGURE 7:**
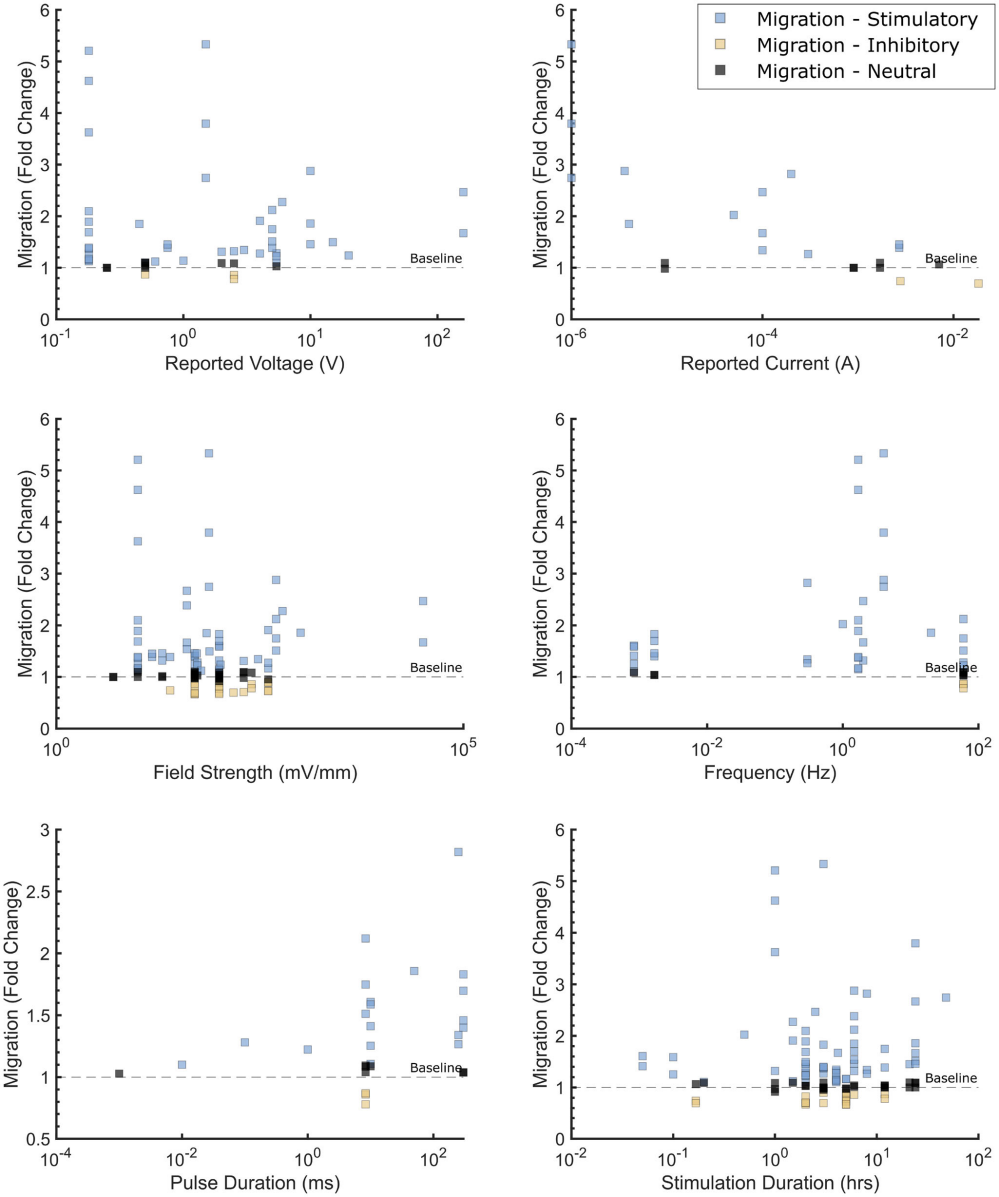
Influence of electrical stimulation parameters on fibroblast migration. Summary of literature‐derived data correlating electrical stimulation parameters (voltage, current, frequency, etc.) with normalized cell migration (fold change). The response profile is color‐coded: Blue data points indicate enhanced migration (>1.1‐fold change), Yellow data points indicate inhibited migration (<0.9‐fold change), and Black data points indicate no significant deviation from the control (0.9–1.1‐fold change). The dashed horizontal line represents the normalized control value of 1.0. Data points were compiled from literature sources listed in the supplementary material.

The corresponding migration data, illustrated in Figure 7, exhibit similar sensitivity to parameter variations. The most pronounced enhancement in migration, quantified as a 5.33‐fold increase, was observed at an applied voltage of 1.5 V [110]. Conversely, inhibitory effects were noted in three instances at voltages of 0.5 and 2.5 V, manifested as a 0.78‐fold change [111]. Voltages below and above the threshold predominantly resulted in neutral to stimulatory effects on migration; for instance, a voltage of 0.25 V resulted in a 1.00‐fold change [28], while a notably high voltage of 160 V induced a substantial increase in migration at 2.47‐fold [98]. These outcomes are likely indicative of factors unique to the specific system under investigation.

Analogous to the effects on proliferation, an increase in the applied current is associated with diminished stimulatory effects. The applied current of 1$\times 10^{-6}$ A resulted in the maximum migration (5.33‐fold increase [110]), whereas a current of 0.0182 A reduced the migration to 0.69‐fold [40].

Optimal electric field strengths were observed to cluster within the range of 10–100 mV/mm, with a field of 75 mV/mm yielding a 5.33‐fold stimulation [110]. In contrast, stronger electric fields, such as 250 mV/mm, resulted in decreased motility (0.67‐fold; [41]).

The modulation of frequency and waveform exhibited a modest effect on the migratory outcomes. A driving frequency of 4 Hz resulted in the highest migration response, quantified as a 5.33‐fold increase [110], whereas at 60 Hz the effects were less stimulatory, with some instances of inhibition marked by a 0.78‐fold change [111]. Extended pulse durations (approximately 250 ms) promoted migration, by a 2.82‐fold enhancement [24], whereas shorter pulses (8.33 ms and below) resulted in reduced stimulatory effects. The notable lack of inhibitory effects across the spectrum of pulse durations is observed, with the exception of pulses at 8.33 ms, which demonstrate a 0.78‐fold alteration in inhibitory response [111]. The trends concerning stimulation duration were consistent, wherein a brief exposure of 3 h elicited the maximal response, a 5.33‐fold increase [110]. Although inhibitory responses were observed within the 0.16 to 12‐h exposure range, both neutral and stimulatory outcomes were also recorded, underscoring the influence of additional parameters on this variable effect.

The cumulative findings of this section show that fibroblast motility is enhanced by low‐intensity, short‐duration electrical stimulation, provided it is confined within a specific electrochemical range. Deviations from this range, specifically through increased current density, negate the beneficial effects, which is likely attributable to alterations in local pH levels and ionic disequilibrium at the electrode–medium interface. Furthermore, apart from the applied current, no singular experimental condition was found to predominantly influence the migratory response.

**FIGURE 8:**
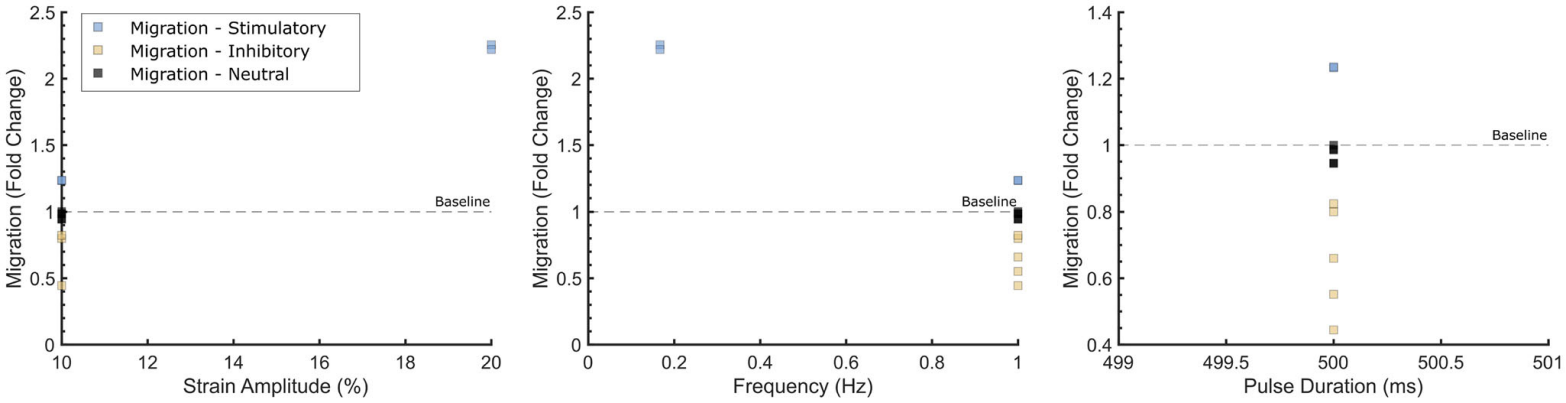
Impact of mechanical stimulation parameters on fibroblast migration. Scatter plots illustrating the normalized migration outcome (fold change) across a range of mechanical stimulation parameters. Each point represents a distinct data point extracted from the literature. The color coding distinguishes the type of cellular response: Blue squares represent pro‐migratory effects (>1.1‐fold change), Yellow squares represent anti‐migratory/inhibitory effects (<0.9‐fold change), and Black squares represent neutral effects (between 0.9 and 1.1‐fold change). The dashed line indicates the unstimulated control baseline (1.0). Data points were compiled from literature sources listed in the supplementary material.

The limited data regarding fibroblast migration in response to mechanical stimuli precludes a definitive data‐driven analysis of this effect (Figure 8). This may be attributable to the practical challenges associated with acquiring migratory data during dynamic stimulation. The lack of reported migration data in existing literature indicates a significant gap in the research. The available data for each single parameter plot is provided in the supplementary material.

#### 4.2.2 Multi‐Parameter Trends

**FIGURE 9:**
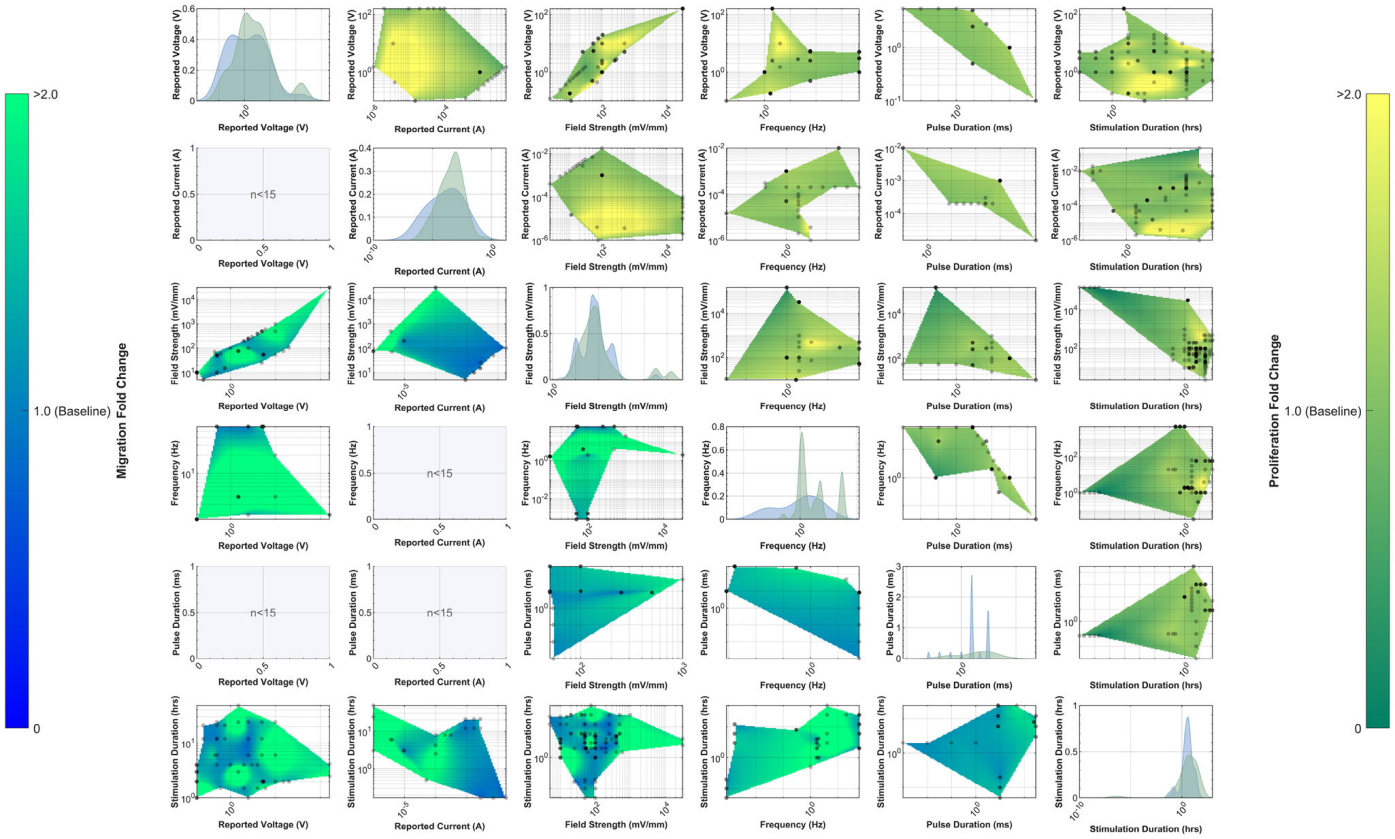
Multiparametric landscape analysis of electrical stimulation parameters on fibroblast behavior. A pairwise comparison matrix visualizing literature‐derived outcomes for Proliferation (green, upper‐right triangle section) and Migration (blue, lower‐left triangle section). The diagonal elements display Kernel Density Estimation (KDE) plots, representing the distribution and density of study parameters reported in the literature. The off‐diagonal plots represent spatially interpolated response surfaces of normalized outcomes (Fold Change relative to control). Individual data points extracted from primary literature are overlaid as black dots to indicate data density while regions without dots represent extrapolated trends. Axes are presented on a logarithmic scale for frequency, voltage, current, and duration parameters to accommodate the wide dynamic range of reported protocols. Color bars represent the normalized fold change relative to baseline (1.0). The color scale is saturated at a 2.0‐fold increase to prevent high‐magnitude outliers from compressing the visual dynamic range, ensuring maximum contrast for biologically significant variations within the 1.0–2.0 range. Background shading indicates the respective outcome zone (Green = Proliferation, Blue = Migration). Data points were compiled from literature sources listed in the supplementary material.

**FIGURE 10:**
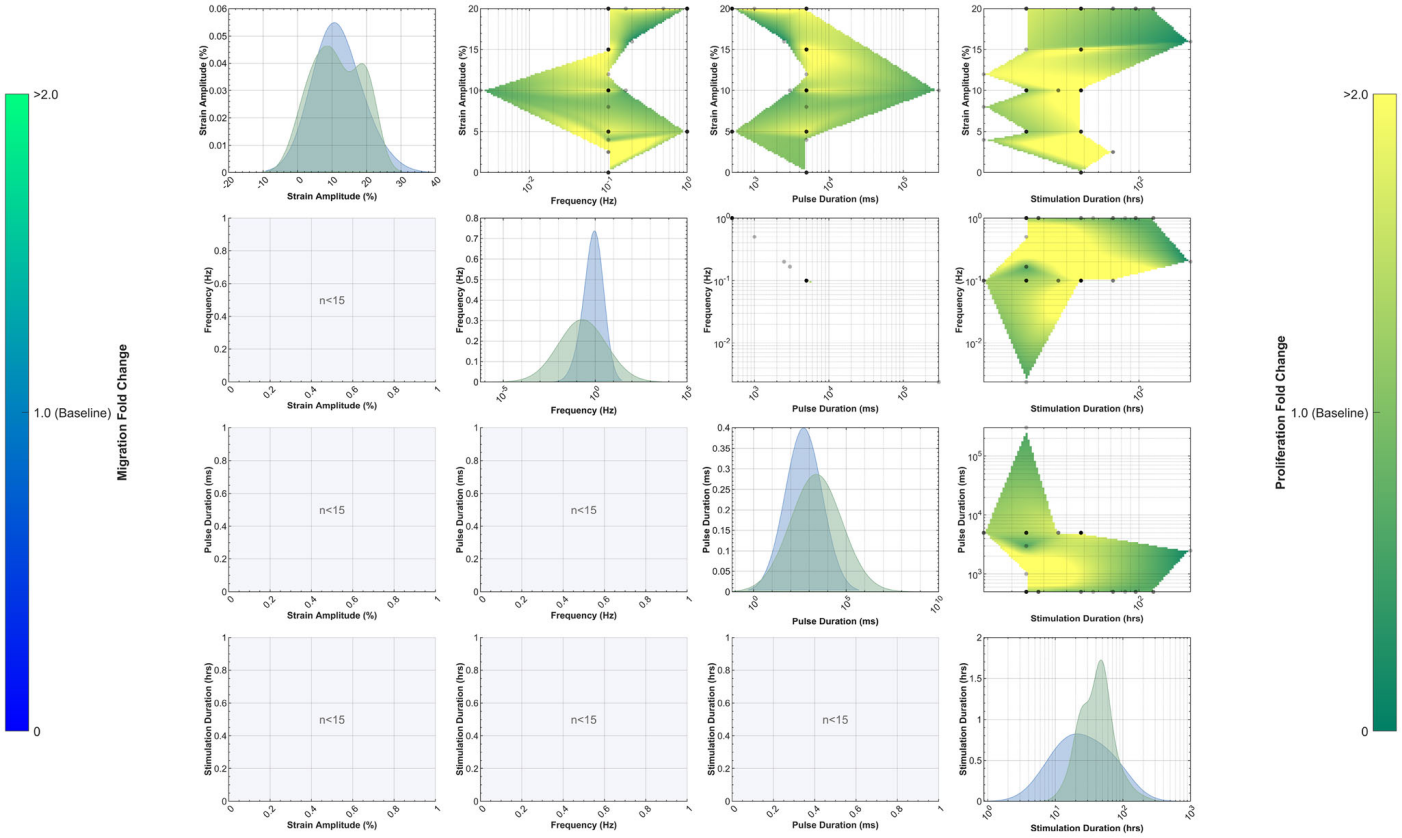
Multi‐parametric landscape analysis of mechanical stimulation parameters on fibroblast behavior. A pairwise comparison matrix visualizing literature‐derived outcomes for Proliferation (green, upper‐right triangle section) and Migration (blue, lower‐left triangle). The diagonal elements display Kernel Density Estimation (KDE) plots, representing the distribution and density of study parameters reported in the literature for proliferation (green fill) and migration (blue fill). Off‐diagonal plots represent spatially interpolated response surfaces of normalized outcomes (Fold Change relative to control). Individual data points extracted from primary literature are overlaid as black dots to indicate data density while parameter pairs with insufficient data (n<5) or lack of variation were excluded to ensure interpolation accuracy. Axes are presented on a logarithmic scale for parameters spanning wide dynamic ranges (e.g., frequency, duration) to facilitate visualization of the protocol landscape. Vertical color bars indicate the normalized fold‐change relative to baseline (1.0). The color scale is saturated at a 2.0‐fold increase to prevent high‐magnitude outliers from compressing the visual dynamic range, ensuring maximum contrast for biologically significant variations within the 1.0–2.0 range. Background shading differentiates the respective outcome zones (Green: Proliferation, Blue: Migration). Data points were compiled from literature sources listed in the supplementary material.

The pairwise parameter interaction matrices for fibroblasts pertaining to electrical stimulation (Figure 9) and mechanical stimulation (Figure 10) are bifurcated such that the panels located in the upper‐right quadrant represent proliferation, whereas those in the lower‐left quadrant are indicative of migration.

The comprehensive analysis of proliferation identified several distinct regions of enhanced stimulation. The relationship between voltage and current demonstrated a 3.24‐fold increase in proliferative response at a voltage of 0.45 V with a current of 4$\times 10^{-6}$ A [105]. Conversely, the same voltage paired with a current of 5.5$\times 10^{-3}$ A resulted in a proliferative inhibition, with a 0.65‐fold response [28]. The analysis of the current–field‐strength map further revealed an optimal proliferative condition near 70–100 mV/mm and 4$\times 10^{-6}$ A, yielding a 3.24‐fold response [81, 105]. In contrast, a significant inhibitory effect was observed at 30 mV/mm when combined with a current of 5.5$\times 10^{-3}$ A, resulting in a 0.65‐fold response [28].

The investigation into field strength–stimulation duration combinations revealed noteworthy results: a 24‐h stimulation at an electric field strength of 100 mV/mm resulted in the highest proliferation rate, with a 3.96‐fold increase [81]. Conversely, ultrashort exposure durations of 1.94$\times 10^{-6}$ h at a field strength of 1.5$\times 10^{5}$ mV/mm reduced proliferation to 0.03‐fold [104]. Intermediate stimulation durations, such as 72 h, led to a moderate enhancement in proliferation, with a 1.17‐fold increase [75]. The impact of the duration is predominantly influenced by the intensity of the applied field, wherein lower intensity fields tend to induce either stimulatory or neutral effects over extended durations.

The interaction of frequency with voltage and field strength exhibited a consistent pattern. At a low frequency of 1.69 Hz with an applied voltage of 0.18 V, a 2.48‐fold amplification was observed [43]. Conversely, a frequency of 1 Hz in conjunction with a high electric field strength of 1.5$\times 10^{5}$ mV/mm resulted in an almost complete suppression, quantified as a 0.03‐fold change [104]. In contrast, at higher frequencies of 4.8$\times 10^{3}$ Hz paired with an applied voltage of 1 V, the outcome was neutral, reflected by a 1.00‐fold change [112].

Pulse‐duration interactions were predominantly influenced by regimes at the millisecond scale. Specifically, a stimulation pulse of 1000 ms duration at a voltage of 1 V resulted in a 1.61‐fold increase [107]. Conversely, shorter pulses of 8 ms, under similar electrical conditions, resulted in effects that were either neutral or inhibitory, manifesting as a 0.94‐fold change [113]. Furthermore, the application of these prolonged pulses in conjunction with a 24‐h stimulation period perpetuated the stimulatory effect, maintaining the same 1.61‐fold increase [107].

Collectively, the fibroblast data confirm that maximal proliferation emerges within a low‐voltage (1—2 V), low‐current (4$\times 10^{-6}$ A) window combined with field strengths around 100 mV/mm and exposure durations of roughly 24 h. Any increase in voltage or current beyond these limits consistently transitions the outcome from stimulatory to inhibitory.

The migration responses demonstrated overlapping yet slightly shifted optimal conditions. The most pronounced synergy was observed in the voltage‐field interaction, wherein a voltage of 1.5 V combined with an electric field strength of 75 mV/mm yielded the maximum enhancement in migration, quantified as a 5.33‐fold increase [110]. When the voltage was elevated to 2.5 V or the electric field strength was increased to 250 mV/mm, the migration rate decreased to 0.78‐fold [111]. Additionally, current‐field pairings revealed migratory hotspots. The same 5.33‐fold increase in migration was reported with a current of 1$\times 10^{-6}$ A and an electric field strength of 75 mV/mm [110], whereas a current of 0.0182 A combined with an electric field strength of 100 mV/mm resulted in a reduction of migration to 0.69‐fold [40]. Throughout the heat map, a distinct inhibitory effect is apparent at higher current amplitudes, even when similar electric fields are applied, underscoring the predominance of this parameter.

The investigation into frequency and stimulation duration pairings revealed a stimulatory zone at an application of 4 Hz for a duration of 3 hours which yielded the largest enhancement in migration, at a 5.33‐fold increase [110]. Conversely, increasing the frequency to 60 Hz or extending the duration to 12 hours resulted in a reduction of motility back to baseline [28]. Notably, even with prolonged exposure (48 h) under moderate electric fields of 100 mV/mm, there was a sustained increase in migration, observed as a 2.74‐fold increase [28]. This suggests that both frequency and duration affect migratory responses, with other contributing conditions needed for consideration to yield maximal results.

The multi‐parameter heat maps and pairwise matrices reveal that fibroblast proliferative responses are strongly context‐dependent: same strain amplitude or pulse duration can be either stimulatory or inhibitory depending on concurrent frequency and exposure time. The dominant stimulatory pocket centers on modest cyclic strain (2.5%) and low frequency (0.1 Hz) producing a five‐fold increase in proliferation. This optimum is shown repeatedly in pairwise combinations where the strain‐frequency‐stimulation time at 2.5%, 0.1 Hz and 72 h gives a 7.08 fold increase [54]. These pairwise maxima indicate a multi‐parameter influence of low cyclic rate and moderate strain amplitude.

Conversely, a distinct inhibitory region emerges with the most extreme suppression (0.10‐fold) when moderately high strain (16%) is paired with elevated pulse durations and very long exposures (pulse 2500 ms, 192 h [55]). This clustering implies a non‐linear, cumulative adverse effect from sustained, higher‐amplitude mechanical loading. Even under conditions of large applied strain, it is possible to induce stimulatory effects by adjusting the frequency, pulse duration, and duration of stimulation. This is evidenced by experimentation conducted at a strain amplitude of 20%, a frequency of 0.1 Hz, a pulse duration of 50 000 ms, and a stimulation period extending over 48 h.

Intermediate zones reveal secondary optima, for example, pulses of 1000 ms combined with high strain (20%) gave moderate stimulation in one pairing (4.00‐fold [108]), whereas very long pulse or extreme pulse‐frequency combinations lead to midline responses (e.g., 5000 ms pulse, 48 h exposure 1.00‐fold [109]). The pulse‐frequency heat map also shows that a pulse duration of 1000 ms at a moderate low frequency of 0.5 Hz resulted in a increase in proliferation. Conversely, slightly elevated cyclical frequencies or ultralow frequencies appear to suppress this effect. For instance, a pulse duration of 2500 ms at a frequency of 0.2 Hz led to a marked inhibitory outcome [55].

Taken together, fibroblast migration is optimized by coordinated parameter combinations involving low current densities (10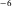 A), moderate field strengths (50–100 mV/mm), and short stimulation durations (2–6 h). Beyond these thresholds, inhibitory effects dominate, likely due to electrochemical and ionic stress. This is also reflected mechanically, where the fibroblast proliferation landscape is most prominent in the literature at low frequency, moderate strain, intermediate exposure times, while the inhibitory effect often arises from combinations of high strain, long pulses and prolonged exposure.

### 4.3 Keratinocyte Stimulation

#### 4.3.1 Survival and Proliferation ‐ Single Parameter Trends

**FIGURE 11:**
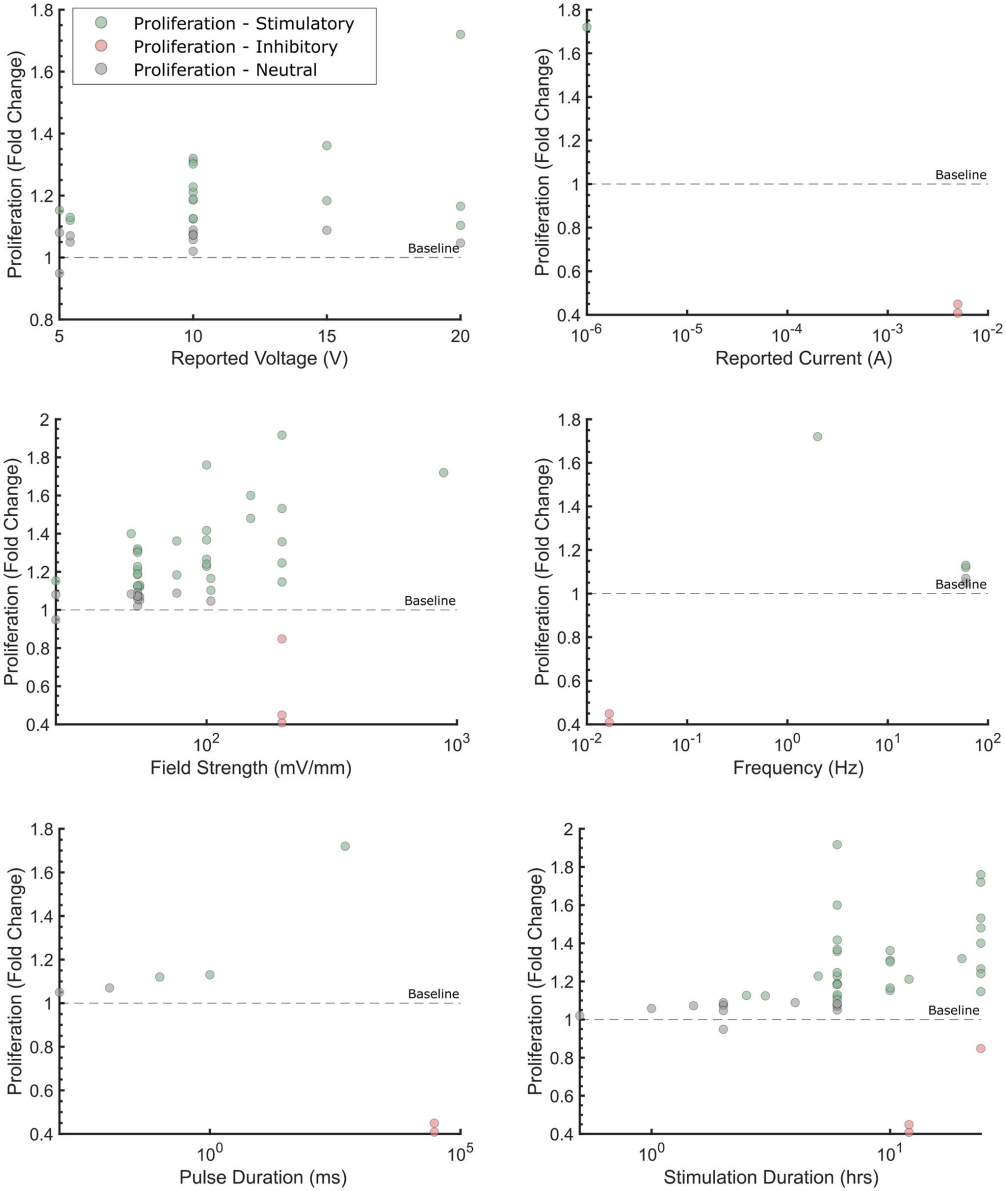
Global parameter analysis of electrical stimulation effects on keratinocyte proliferation. Comprehensive visualization of normalized proliferative outcomes plotted against key electrical stimulation parameters, including reported voltage, current, field strength, frequency, and pulse duration. Data points are color‐coded to visualize efficacy: Green indicates a stimulatory proliferative response (>1.1‐fold change), Red indicates inhibition (<0.9‐fold change), and Grey indicates a neutral outcome. The dashed line (y=1.0) denotes the control baseline. Parameters with wide variance are plotted on a logarithmic x‐axis to facilitate trend visualization. Data points were compiled from literature sources listed in the supplementary material.

The proliferation of keratinocytes exhibited a more constrained range of stimulation compared to that of fibroblasts (Figure 11). The maximum proliferative response, quantified as a 1.72‐fold increase, was observed at an electrical potential of 20 V and an electric field strength of 885 mV/mm [9]. In contrast, a relatively low potential of 5 V at an electric field strength of 25 mV/mm resulted in a suppression of proliferation to 0.95‐fold [76]. Conditions that were approximately intermediate, such as those at 10 V and 53 mV/mm, yielded a neutral effect on proliferation, indicated by a 1.02‐fold change [76].

As with fibroblasts, the current markedly affected the experimental outcomes. Specifically, stimulation using microampere currents (1$\times 10^{-6}$ A) resulted in a 1.72‐fold proliferation enhancement [9]. Conversely, the application of higher current levels (0.005 A) was associated with a decrease in cellular proliferation to 0.41‐fold [114]. The investigation into frequency revealed an optimum at low frequencies, where a frequency of 2 Hz was associated with the maximal enhancement [9]. In contrast, very low frequency stimulation at 0.0167 Hz or extended duty cycles exerted an inhibitory effect, reducing the parameter to 0.41‐fold [114], while frequencies around 60 Hz, exhibited a neutral effect on the outcomes, with a change of 1.05‐fold [44].

Proliferation was affected by the pulse width and duration. Intermediate pulse durations of 500 ms facilitated cellular proliferation, showing a 1.72‐fold increase [9]. In contrast, exceedingly long pulse durations of 30 000 ms or sustained stimulation over 12 h resulted in a decline in activity to 0.41‐fold [114]. Conversely, brief pulse durations of 0.001 ms and short exposure times of 0.5 h had negligible effects, maintaining a neutral activity at 1.0‐fold [76]. However, a 6‐h stimulation at an electric field strength of 200 mV/mm effectively sustained activity, resulting in a 1.92‐fold increase [63].

**FIGURE 12:**
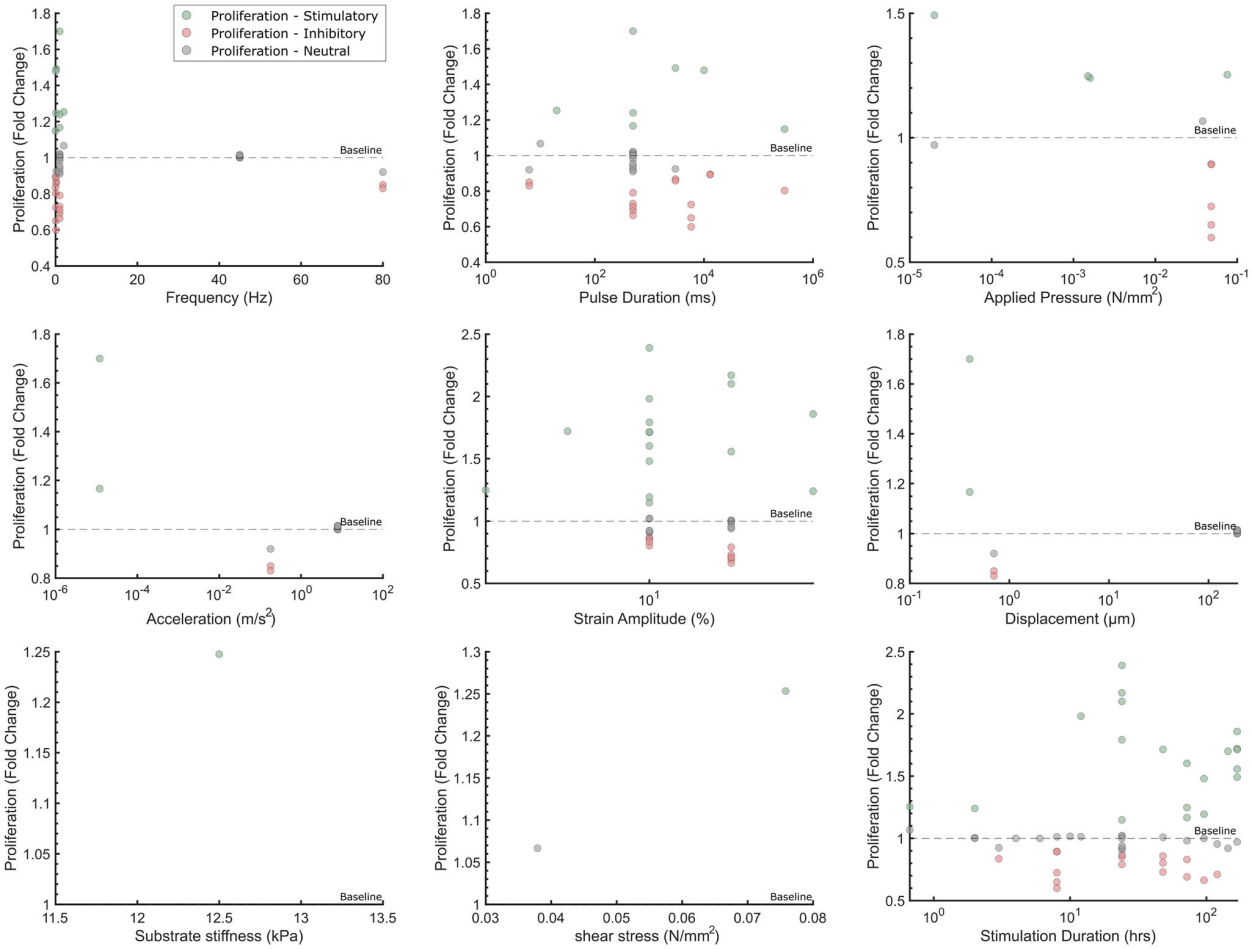
Global analysis of mechanical stimulation parameters on keratinocyte proliferation. Aggregated literature data displaying the normalized proliferative outcome (fold change relative to unstimulated controls) as a function of various mechanical parameters, including displacement, strain amplitude, frequency, and substrate stiffness. Data points represent individual experimental outcomes. Colors denote the nature of the response based on a defined threshold: Green circles indicate a stimulatory effect (>1.1‐fold change), Red circles indicate an inhibitory effect (<0.9‐fold change), and Grey circles indicate a neutral response (0.9–1.1‐fold change). The dashed horizontal line at y=1.0 represents the baseline control value. X‐axes are presented on a logarithmic scale where data ranges span multiple orders of magnitude. Data points were compiled from literature sources listed in the supplementary material.

The keratinocyte dataset for mechanical stimulation is smaller but consistent in showing an optimum mid‐range mechanical input (Figure 12). Surface displacement and acceleration experiments indicate that small micrometric displacements (0.4 μm) and tiny accelerations (1.2$\times 10^{-5}$ m $s^{-2}$) produced modest stimulation of proliferation (1.70‐fold) [69]. Strain amplitude also shows a stimulatory peak at 10 % (2.39‐fold) with 24 h exposure (2.39‐fold) [59], while higher amplitudes (20–40) can be neutral or inhibitory depending on other settings (0.66–1.24 range).

Though limited, shear and applied pressure metrics give corroborating evidence: localised pressure/shear of 0.0758 N ${\mathrm{mm}}^{-2}$ was stimulatory (1.25‐fold) [49], and substrate stiffness of 12.5 kPa supported a similar modest increase (1.25‐fold) [54]. Pulse and frequency effects align with this middle‐ground behavior, 1 Hz drives and 500 ms pulses were associated with the larger keratinocyte proliferative responses (1.70‐fold) [69], whereas low frequency extremes or unusual long pulses (e.g. 5.81$\times 10^{3}$ ms) resulted in more inhibitory outcomes (0.60‐fold) [115].

The aggregated data suggest that keratinocytes exhibit a lower tolerance to over‐stimulation compared to fibroblasts. Electrically, they demonstrate optimal responsiveness to low‐current, moderate‐field, and short‐duration regimens that emulate constant direct current (DC) exposure, particularly within the voltage range of 5–20 V and an electric field strength of 100–200 mV/mm. Mechanically, keratinocyte proliferation is preferred at moderate mechanical amplitudes (strain 10%), short‐to‐intermediate pulses (500 ms) and intermediate exposure times (24 h), with both under‐ and over‐stimulation reducing the effect.

#### 4.3.2 Migration ‐ Single Parameter Trends

**FIGURE 13:**
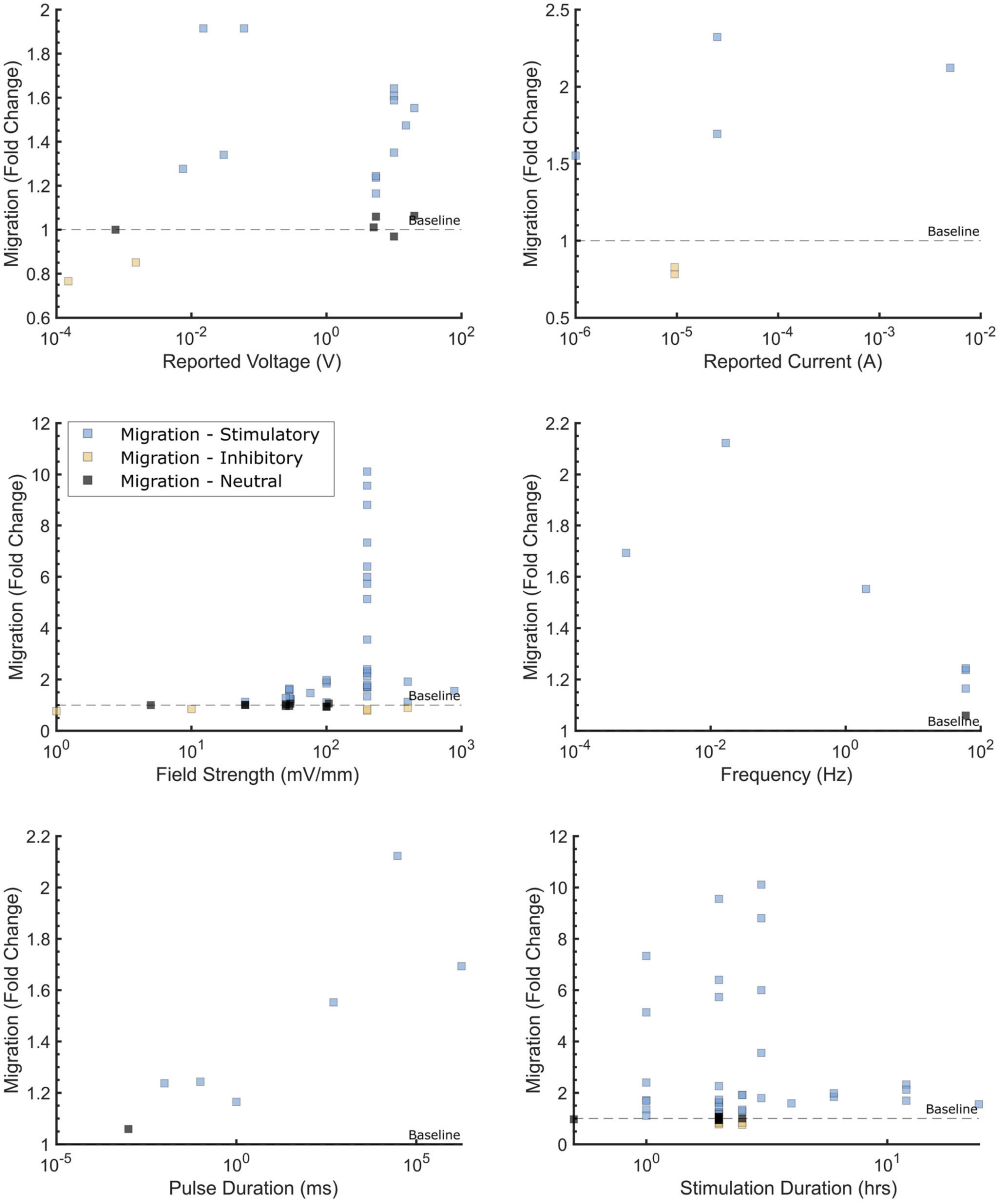
Influence of electrical stimulation parameters on keratinocyte migration. Summary of literature‐derived data correlating electrical stimulation parameters (voltage, current, frequency, etc.) with normalized cell migration (fold change). The response profile is color‐coded: Blue data points indicate enhanced migration (>1.1‐fold change), Yellow data points indicate inhibited migration (<0.9‐fold change), and Black data points indicate no significant deviation from the control (0.9–1.1‐fold change). The dashed horizontal line represents the normalized control value of 1.0. Data points were compiled from literature sources listed in the supplementary material.

The investigation into keratinocyte migration revealed that the most significant augmentations were observed among all evaluated parameters (Figure 13). The maximal response, quantified as a 10.11‐fold increase, was recorded at an electric field strength of 200 mV/mm with a duration of exposure of 3 h [116]. Conversely, extremely weak electric fields, specifically 1 mV/mm, were found to be inhibitory, demonstrated by a 0.77‐fold reduction in migration [117]. Conditions with a moderate field strength of 5 mV/mm were observed to be neutral, maintaining migration at a 1.00‐fold level [117]. Concurrently, stimulation at 0.015 V resulted in a 1.91‐fold enhancement in keratinocyte migration, whereas very low potentials of 0.00015 V reduced migration to 0.77‐fold [117]. The limited number of data points of current stimulation effects on proliferation hinders our ability to draw direct relationships from the small sample size. The frequency and pulse duration of electrical stimulation exhibit nuanced effects on keratinocyte motility. Stimulation at a low frequency of 0.0167 Hz resulted in a 2.12‐fold increase in motility [114], whereas stimulation at a frequency of 60 Hz yielded results that were nearly neutral, with a 1.06‐fold change [44]. Long‐duration pulses of 3$\times 10^{4}$ ms demonstrated a similar stimulatory effect, showing a 2.12‐fold increase [114], whereas very brief pulses of 0.001 ms were neutral, with a 1.06‐fold effect noted by Li et al. [44]. Shorter treatments of around 3 hours produced the most significant increases in motility. Conversely, prolonged exposure or treatments involving high current intensity tended to attenuate the stimulatory effect.

**FIGURE 14:**
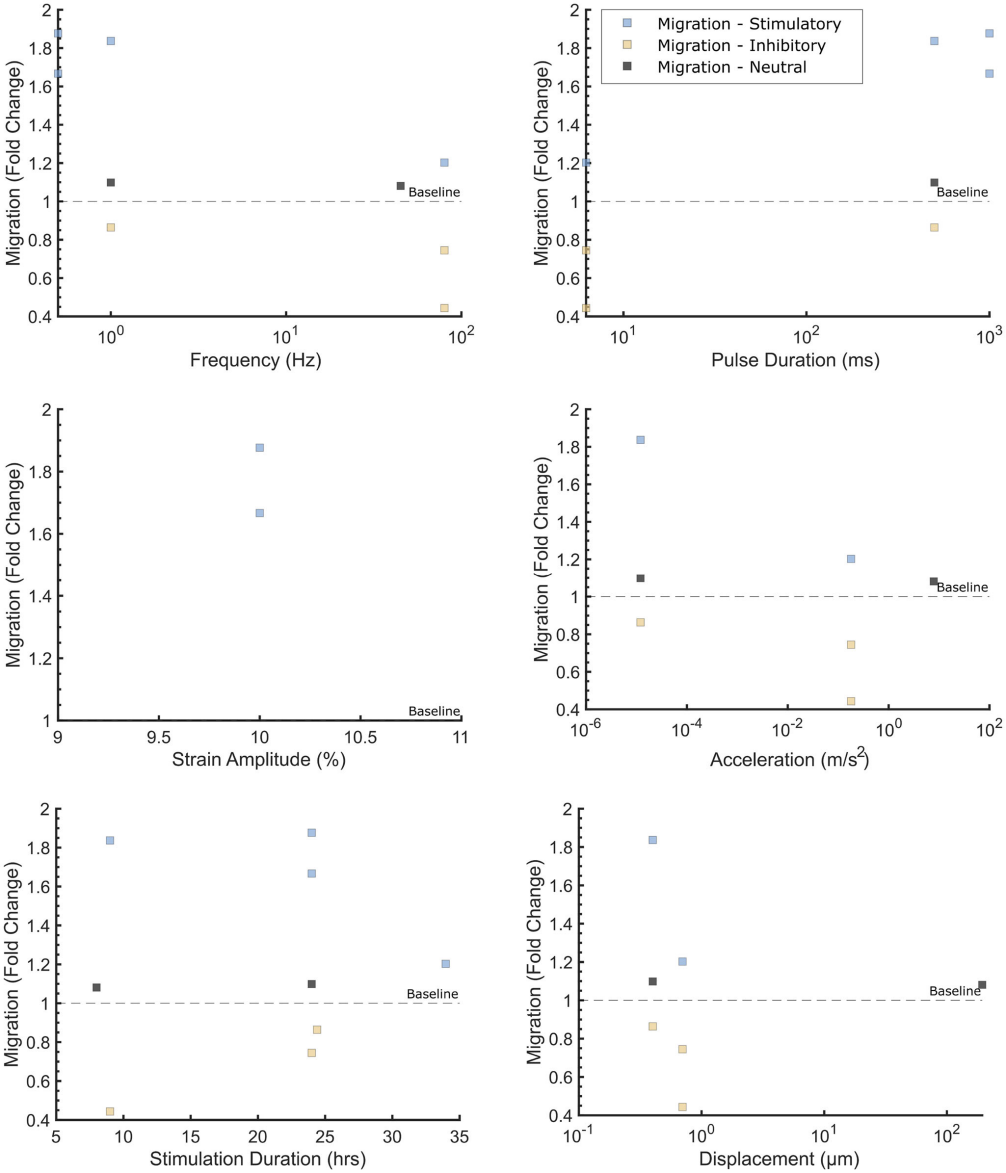
Impact of mechanical stimulation parameters on keratinocyte migration. Scatter plots illustrating the normalized migration outcome (fold change) across a range of mechanical stimulation parameters. Each point represents a distinct data point extracted from the literature. The color coding distinguishes the type of cellular response: Blue squares represent pro‐migratory effects (>1.1‐fold change), Yellow squares represent anti‐migratory/inhibitory effects (<0.9‐fold change), and Black squares represent neutral effects (between 0.9 and 1.1‐fold change). The dashed line indicates the unstimulated control baseline (1.0). Data points were compiled from literature sources listed in the supplementary material.

Similar to the fibroblast migration data for mechanical stimulation, significantly fewer reports were available for analysis (Figure 14). Few studies suggest that small displacements (0.4 μm) and very low accelerations can produce migratory stimulation (1.84‐fold) [69]. Strain amplitude around 10% was again favourable (1.88‐fold) [32], whereas higher frequencies (80 Hz) or very small pulse widths (6.25 ms) were inhibitory (0.44‐fold) [69]. The largest migration responses were observed with low frequency cyclical loading (0.5 Hz), long pulses (1000 ms) at 24 h dosing (1.88‐fold across these conditions [32]). Further data experimental data collection for the mechanical effects on keratinocyte migration is required before a definitive conclusion can be drawn. The available data for each single parameter plot is provided in the supplementary material.

Overall, keratinocytes demonstrate a conserved electrotactic optimum characterized by weak, steady electric and strain fields with low current densities and displacements. The reproducibility of this window across independent studies suggests that keratinocyte migration is sensitive to deviations in both field strength and exposure time. The insufficient documentation of inhibitory migration responses may introduce bias into the observed trends, thereby creating gaps in the literature. This scarcity of data complicates the selection of stimulation parameters based on extant studies.

#### 4.3.3 Multi‐Parameter Trends

**FIGURE 15:**
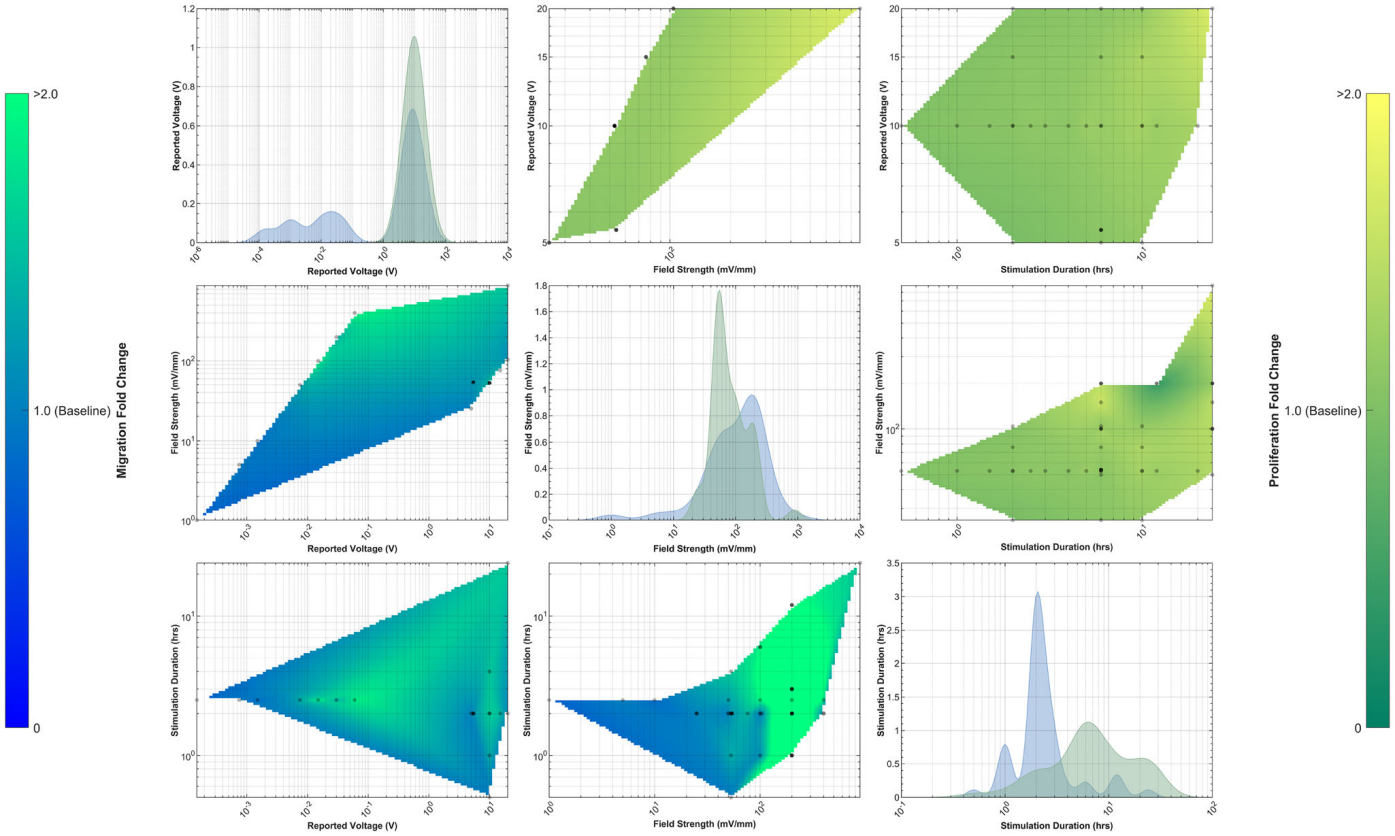
Multiparametric landscape analysis of electrical stimulation parameters on keratinocyte behavior. A pairwise comparison matrix visualizing literature‐derived outcomes for Proliferation (green, upper‐right triangle section) and Migration (blue, lower‐left triangle section). The diagonal elements display Kernel Density Estimation (KDE) plots, representing the distribution and density of study parameters reported in the literature. The off‐diagonal plots represent spatially interpolated response surfaces of normalized outcomes (Fold Change relative to control). Individual data points extracted from primary literature are overlaid as black dots to indicate data density while regions without dots represent extrapolated trends. Axes are presented on a logarithmic scale for frequency, voltage, current, and duration parameters to accommodate the wide dynamic range of reported protocols. color bars represent the normalized fold change relative to baseline (1.0). The color scale is saturated at a 2.0‐fold increase to prevent high‐magnitude outliers from compressing the visual dynamic range, ensuring maximum contrast for biologically significant variations within the 1.0‐2.0 range. Background shading indicates the respective outcome zone (Green = Proliferation, Blue = Migration). Data points were compiled from literature sources listed in the supplementary material.

The electrical stimulation heat map matrix for keratinocytes, illustrated in Figure 15, comprised of a reduced set of complete parameter pairs, limiting extensive multi‐parameter analysis.

The interaction between voltage and electric field revealed that a parameter set of 20 V and 885 mV/mm resulted in a 1.72‐fold increase in proliferation [9], whereas conditions of 5 V and 25 mV/mm led to a diminished proliferation rate, a 0.95‐fold change [76]. Conditions with intermediate values, approximately 10 V and 53 mV/mm, demonstrated a neutral effect, with a proliferation change of 1.02‐fold [76]. A similar trend was observed in the duration‐dependent data; specifically, a 6‐h exposure at 200 mV/mm yielded a 1.92‐fold enhancement in proliferation [63], whereas a 12‐h exposure under the same electric field strength resulted in an inhibitory effect, with a decrease to 0.41‐fold [114].

Migration data displayed more pronounced multi‐parametric interplay. The field‐voltage map showed maximal migration at 0.015 V and 100 mV/mm (1.91‐fold [117]), compared with inhibition at 1.5 $\times 10^{-5}$ V and 1 mV/mm (0.77‐fold [117]). The highest recorded keratinocyte migration overall occurred at 200 mV/mm with 3 h stimulation (10.11‐fold [116]). The prevalence of migration hotspots is notably higher when assessing the effects of field strength and stimulation duration. Enhanced motility is observed at elevated field strengths, independent of the stimulation duration. Conversely, extending the stimulation duration at a constant field strength tends to diminish the response.

Despite their absence from the heat maps due to a scarcity of data points, the current–duration interaction revealed that maintaining a current of 2.5$\times 10^{-5}$ A for a duration of 3 hours resulted in a 2.32‐fold increase in motility [116, 118]. Conversely, decreasing the current to 9.5$\times 10^{-6}$ A or prolonging the exposure beyond 6 hours inhibits the response, resulting in a 0.78‐fold change [39, 114]. Furthermore, low‐frequency, long‐duration pulse combinations (specifically 0.0167 Hz and 3$\times 10^{4}$ ms) were found to enhance motility, as evidenced by a 2.12‐fold increase [114]. In contrast, pulses of 0.001 ms at a frequency of 60 Hz exhibited a neutral effect, with a 1.06‐fold change [44].

**FIGURE 16:**
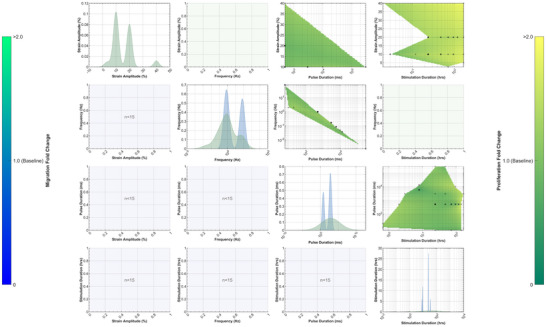
Multiparametric landscape analysis of mechanical stimulation parameters on keratinocyte behavior. A pairwise comparison matrix visualizing literature‐derived outcomes for Proliferation (green, upper‐right triangle section) and Migration (blue, lower‐left triangle). The diagonal elements display Kernel Density Estimation (KDE) plots, representing the distribution and density of study parameters reported in the literature for proliferation (green fill) and migration (blue fill). Off‐diagonal plots represent spatially interpolated response surfaces of normalized outcomes (Fold Change relative to control). Individual data points extracted from primary literature are overlaid as black dots to indicate data density while parameter pairs with insufficient data (n<5) or lack of variation were excluded to ensure interpolation accuracy. Axes are presented on a logarithmic scale for parameters spanning wide dynamic ranges (e.g., frequency, duration) to facilitate visualization of the protocol landscape. Vertical color bars indicate the normalized fold‐change relative to baseline (1.0). The color scale is saturated at a 2.0‐fold increase to prevent high‐magnitude outliers from compressing the visual dynamic range, ensuring maximum contrast for biologically significant variations within the 1.0–2.0 range. Background shading differentiates the respective outcome zones (Green: Proliferation, Blue: Migration). Data points were compiled from literature sources listed in the supplementary material.

Keratinocyte pairwise heat maps for mechanical stimulation (Figure 16) reinforce a mid‐range mechanosensitivity: strain amplitude 10%, pulse durations at $\times 10^{4}$ ms and 24 h exposures provided the maximal stimulation (2.39 fold [59]). Multi‐parameter cluster show variable results: pulse duration‐strain shows stimulatory pockets at high and low pulse lengths with strain 10% strain at $\times 10^{4}$ ms and a 40% strain at 500 ms yielding stimulatory outcomes. This is supported at from the pulse‐frequency map where a 500 ms pulse at 1 Hz also aligns with a local maximum (1.70‐fold) [69]. Stimulation duration‐strain maps reveal a distinct maximum at 24 hours and 10% strain, showing a 2.39‐fold increase [59]. Deviations from this duration, either longer or shorter, tend to result in a neutral or inhibitory response until reaching a 72‐hour time point, beyond which an extended duration engenders a proliferative outcome across the range of strains analysed.

For mechanical stimuli, unlike fibroblasts, keratinocyte outcomes show fewer extremes in the compiled dataset (i.e,. fewer multi‐log fold changes), but the same principle holds: strain, pulse and stimulation duration combined control the stimuli response. Notably, the analysis of pulse‐frequency mappings indicates that stimulatory conditions for proliferation are centered around moderate pulse durations (500 ms) coupled with low to moderate frequencies (1 Hz) [69]. In contrast, extreme parameter combinations, such as extended pulse durations paired with low frequencies or minimal pulse durations at high frequencies, can exhibit inhibitory effects, as demonstrated by the specific case of a pulse duration of 5.81$\times 10^{3}$ ms at a frequency of 0.086 Hz resulting in 0.60‐fold reduction in proliferation [115].

Combined, the parameter interaction data corroborate that both fibroblasts and keratinocytes exhibit a shared general responsiveness favoring low current densities and moderate field strengths. However, they diverge in their temporal sensitivities, as fibroblasts demonstrate tolerance to longer exposures, whereas keratinocytes attain an optimal response under shorter, weaker stimulation. Furthermore, mechanically, these maps suggest keratinocyte proliferation is governed by a narrower multi‐parameter band than fibroblasts, favoring moderate cyclicity and mid‐amplitude strain.

#### 4.3.4 Proposed Figures of Merit for Electrical Stimulation

The apparent multi‐parametric effects elicited by electrical stimulation raise a critical question: *can the modulation of a singular stimulation parameter reliably predict cellular behavioral changes?* Analysis of the reported data unequivocally demonstrates that each electrical parameter—voltage, current, frequency, pulse duration, and cumulative duration of stimulation—does not always have the capacity to independently affect cellular responses. Thus, designating any singular variable as a universal control parameter fails to adequately capture the intricate nature of the system.

A more comprehensive approach is therefore required, one that integrates multiple stimulation settings into composite metrics capable of representing the overall energetic or electrochemical load experienced by the cells. Three potential quantitative descriptors emerge as candidates for such integrative analysis: (1) *power*–the instantaneous rate of energy delivery; (2) *total energy delivered*–the cumulative energy imparted to the system over the stimulation period; and (3) *total charge delivered*–the sum of charge transferred across the electrodes during stimulation.

**FIGURE 17:**
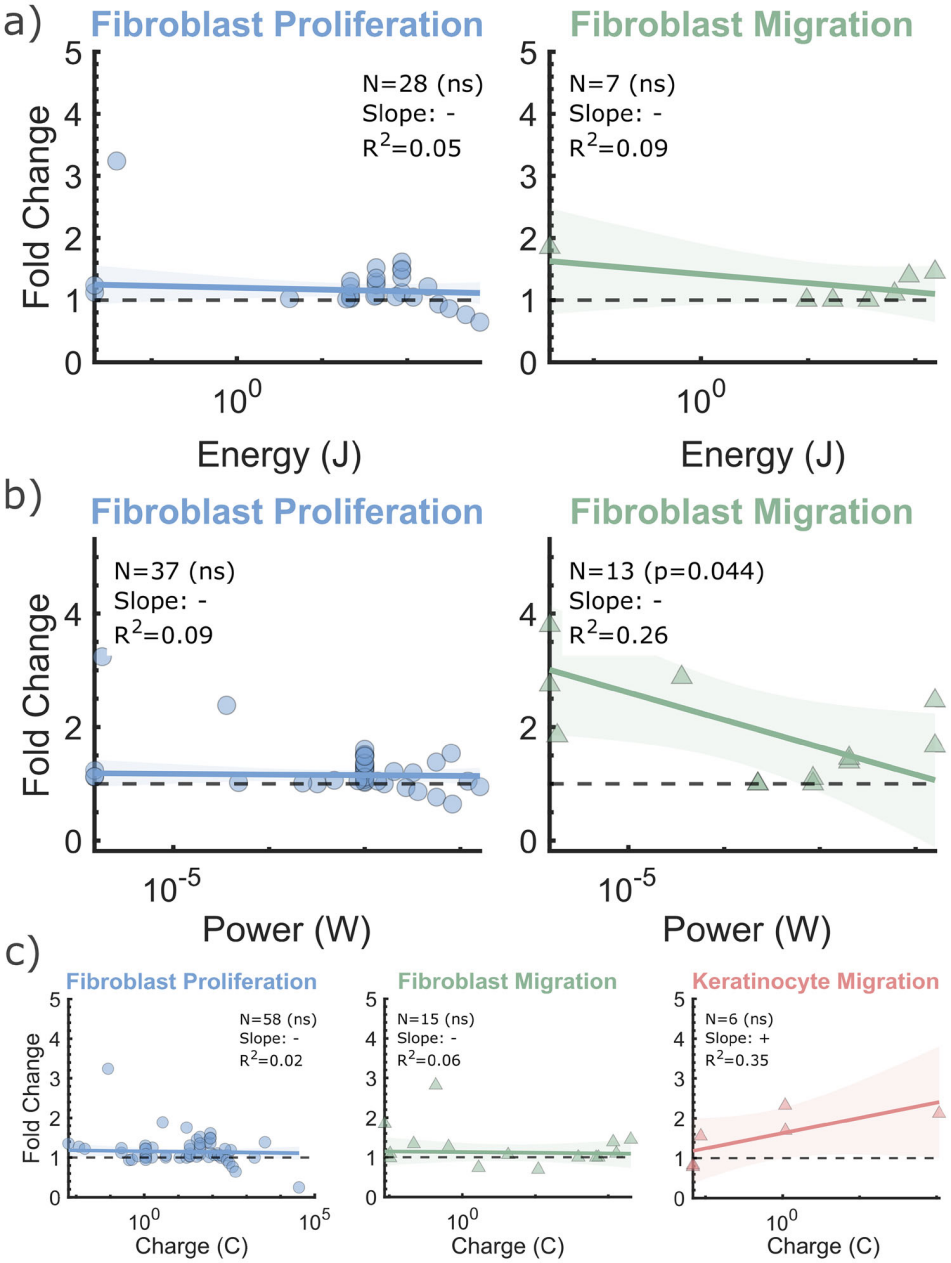
Comparison of the three integrated stimulation metrics–(a) power, (b) total energy delivered, and (c) total charge delivered‐showing their respective relationships with the observed cellular responses. Each plot visualises the combined effect of multiple stimulation parameters on fibroblast proliferation and migration outcomes. Data points were compiled from literature sources listed in the supplementary material.

In this dataset, we evaluated power, energy, and charge as candidate composite stimulation metrics, each intrinsically integrating voltage, current, and time. However, none of these parameters, when considered in isolation, reliably predicted fibroblast or keratinocyte behavior (Figure 17). For fibroblast proliferation, power (Adj. $R^{2}$ = 0.09), energy (Adj. $R^{2}$ = 0.06), and charge (Adj. $R^{2}$ = 0.02) exhibited only weak associations, despite spanning multiple orders of magnitude (10
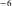
–10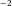 W, 10
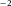
–10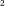 J, and 10
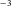
–10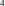 C, respectively) and encompassing strongly stimulatory (fold change > 3), neutral, and inhibitory responses. Migration outcomes were similarly inadequately described, with fibroblast migration showing only modest to weak relationships with power (Adj. $R^{2}$ = 0.26), energy (Adj. $R^{2}$ = 0.09), and charge (Adj. $R^{2}$ = 0.06), while keratinocyte migration demonstrated a moderate but statistically uncertain association as a function of charge (Adj. $R^{2}$ = 0.35). Collectively, these findings suggest that although power, energy, and charge each incorporate core electrical variables (voltage, current, and time), no single one‐dimensional metric is sufficient to characterize the effective electrical dose. A more appropriate descriptor for skin cell stimulation will likely require the simultaneous integration of multiple electrical parameters, including their temporal patterning, rather than reduction to a single scalar quantity. It should also be noted that, due to the limited number of literature publications providing full characterization of the stimulation parameters, only a small sample (6–58) of data points was available for this analysis, which may skew the resulting trends.

While composite metrics such as power, energy, and charge currently fail to provide a universal predictor for cellular behavior, this inconsistency may be compounded by the high degree of “noise” in existing literature. Disparities in electrode materials, stimulation geometries, and reporting standards make meta‐analysis across distinct studies challenging.

To resolve this, we recommend that future research focuses on isolated studies using standardized cell lines to untangle the specific effects of combined stimulation parameters. If simple metrics remain insufficient, the field should move toward weighted parametric formulations. By mathematically combining field strength, frequency, and duration into a unified ‘stimulation dose– metric, researchers can establish a more reliable framework for cross‐study comparison and predictive control over cellular responses such as proliferation and migration.

## 5 Conclusions

This data‐driven review synthesizes current knowledge on cellular responses and underlying mechanisms elicited by electrical and mechanical stimulation, based on the available literature. We present an analysis of the in vitro effects of electrical and mechanical stimulation at the cellular level, encompassing alterations in cell behavior (proliferation and migration), intracellular processes (protein activation and redistribution), and extracellular organisation (ECM remodeling).

By evaluating a standardized dataset of migration and proliferation measurements, we show that, when considered as an isolated variable, the applied current in electrical stimulation exerts an inhibitory effect on cell proliferation and migration. Only when multi‐parameter relationships are examined do combined parameter effects emerge, supporting the data‐driven hypothesis that modulation of in vitro behavior depends on complex interactions among stimulation parameters.

When stimulation parameters are assessed in a multivariate context (power, total delivered energy, and total delivered charge), an emerging, albeit weak, trend is observed: fibroblast migration and proliferation decrease with increasing power, energy, and charge, whereas keratinocytes exhibit an increase in migration with increasing charge. The scarcity of datasets that comprehensively report multiple stimulation parameters precludes the establishment of definitive conclusions. However, this lack of data underscores the necessity for standardized experimental studies featuring rigorous characterization of electrical stimulation configurations and an exhaustive sweep of stimulation parameters to fully elucidate the causal relationships underlying these multiparametric interactions.

Mechanical stimulation studies similarly report both stimulatory and inhibitory outcomes across different strain magnitudes, loading rates, and stimulation durations. However, the limited availability of datasets that systematically vary multiple mechanical parameters constrains direct cross‐comparisons, despite evident variability in cellular responses. We postulate that a comparable multiparameter optimization framework for both electrical and mechanical stimulation, including an exhaustive study of mechanical stimulation regimes, is needed to achieve a more complete understanding of the relationships and to enable more precise and predictable control of cell behavior.

## 6 Data Methodology

### 6.1 Literature Search Strategy

A search was conducted using Web of Science for all articles published up to September 1, 2025. The search strategy focused on specific cell types “Fibroblast” and “Keratinocyte,” filtering papers by the type of stimulation (“electrical stimulation,” “mechanical stimulation,” “electro‐mechanical stimulation” and “mechano‐electric stimulation”) across the abstract, keywords and main text. References included in each article were reviewed manually and included if identified as eligible. We considered articles eligible if they reported data in which an in vitro study included a stimulated group with at least one of the typical electrical and/or mechanical stimulation parameters (see Table 1) that was directly comparable to a non‐stimulated control group in an original peer‐reviewed research article.

Studies were excluded if they were review articles, reports or only presented in vivo and clinical studies or did not provide stimulation parameters. Those that did not contain date along side a relevant control were also dismissed from the analysis.

### 6.2 Data Extraction and Normalization

Electrical and mechanical stimulation parameters, as summarised in (Table 1) were extracted from the compiled literature using PlotDigitizer (https://plotdigitizer.com/). From each study, stimulation parameters were collected alongside the measured biological outcomes (e.g., proliferation or migration) and the corresponding quantitative metric reported from the in vitro assay. For each dataset, both stimulated and control values were extracted to calculate a normalized outcome, expressed as a fold change relative to the control condition.

In addition to the stimulation parameters, the cell type, cell line, and source were recorded for all studies and systematically tabulated. Cell types were standardised through keyword‐based classification, grouping entries containing “Fibroblast” or “HDF” under the “Fibroblast Group” and those containing “Keratinocyte” under the “Keratinocyte Group”. Entries not matching these criteria were classified as “Other” and excluded from the primary analysis. The complete tabulated dataset, including all extracted and normalized values, is provided in the Supporting Information.

### 6.3 Data Analysis and Grouping

The normalized data were analysed in MATLAB (MathWorks, Natick, MA) using a custom processing pipeline. The dataset was first divided by cell group (Fibroblast and Keratinocyte) and subsequently by outcome type (Proliferation or Migration). Each subgroup was processed independently to evaluate parameter–response relationships.

Within each subgroup, outcome data were visualized using scatter plots, box plots, and heatmaps to identify parameter trends. Fold‐change values were categorised as stimulatory (>1.1×), inhibitory (<0.9×), or neutral (0.9–1.1×) relative to control. Further statistical and graphical analyses were conducted to examine potential parameter interactions, including multi‐parameter heatmaps, distribution plots, and literature density matrices.

Comprehensive data tables summarizing the extracted parameters and normalized outcomes are provided in the Supporting Information.

## Conflicts of Interest

The authors declare no conflicts of interest.

## Supporting information


**Supporting File**: adhm71138‐sup‐0001‐Data.zip.

## Data Availability

For the purpose of Open Access, the authors have applied a CC BY public copyright licence to any Author Accepted Manuscript (AAM) version arising from this submission. No original data were generated as part of this study. However, the data used to generate the comparisons in this work are available in the supporting information. An open, community‐extensible dataset and interactive visualization dashboard is accessible at data.smart‐biomaterials.com/stim‐dashboard, where researchers can contribute additional data via pull request.
